# High Diversity of Anaerobic Alkane-Degrading Microbial Communities in Marine Seep Sediments Based on (1-methylalkyl)succinate Synthase Genes

**DOI:** 10.3389/fmicb.2015.01511

**Published:** 2016-01-07

**Authors:** Marion H. Stagars, S. Emil Ruff, Rudolf Amann, Katrin Knittel

**Affiliations:** ^1^Department of Molecular Ecology, Max Planck Institute for Marine MicrobiologyBremen, Germany; ^2^HGF MPG Joint Research Group for Deep-Sea Ecology and Technology, Max Planck Institute for Marine MicrobiologyBremen, Germany

**Keywords:** alkyl succinate synthase, MasD, AssA, sulfate reduction, anaerobic alkane oxidation, microbial diversity, geneFISH

## Abstract

Alkanes comprise a substantial fraction of crude oil and are prevalent at marine seeps. These environments are typically anoxic and host diverse microbial communities that grow on alkanes. The most widely distributed mechanism of anaerobic alkane activation is the addition of alkanes to fumarate by (1-methylalkyl)succinate synthase (Mas). Here we studied the diversity of MasD, the catalytic subunit of the enzyme, in 12 marine sediments sampled at seven seeps. We aimed to identify cosmopolitan species as well as to identify factors structuring the alkane-degrading community. Using next generation sequencing we obtained a total of 420 MasD species-level operational taxonomic units (OTU_0.96_) at 96% amino acid identity. Diversity analysis shows a high richness and evenness of alkane-degrading bacteria. Sites with similar hydrocarbon composition harbored similar alkane-degrading communities based on *MasD* genes; the MasD community structure is clearly driven by the hydrocarbon source available at the various seeps. Two of the detected OTU_0.96_ were cosmopolitan and abundant while 75% were locally restricted, suggesting the presence of few abundant and globally distributed alkane degraders as well as specialized variants that have developed under specific conditions at the diverse seep environments. Of the three MasD clades identified, the most diverse was affiliated with Deltaproteobacteria. A second clade was affiliated with both Deltaproteobacteria and Firmicutes likely indicating lateral gene transfer events. The third clade was only distantly related to known alkane-degrading organisms and comprises new divergent lineages of MasD homologs, which might belong to an overlooked phylum of alkane-degrading bacteria. In addition, *masD* geneFISH allowed for the *in situ* identification and quantification of the target guild in alkane-degrading enrichment cultures. Altogether, these findings suggest an unexpectedly high number of yet unknown groups of anaerobic alkane degraders and underline the need for comprehensive surveys of microbial diversity based on metabolic genes in addition to ribosomal genes.

## Introduction

Alkanes are found throughout nature and belong to the most abundant organic compounds in the biogeosphere (Wilkes et al., 2002). They are main components of crude oil and natural gas and are either formed by living organisms (Tissot and Welte, 1984) or through geological transformation of biomass (Claypool and Kvenvolden, 1983). In the marine environment, alkanes occur in gas hydrates, petroleum-rich hydrothermal sediments, seeps or areas anthropogenically contaminated such as occurred during the *Exxon Valdez* oil spill in 1989 or the *Deep Water Horizon* blowout in 2010. Marine seeps can differ remarkably in their hydrocarbon composition and concentration. Methane seeps mainly emit methane in micromolar to millimolar ranges m^-2^ d^-1^ (e.g., Hydrate Ridge in the NE Pacific, Suess et al., 1999), gas seeps emit a substantial amount of C_2_-C_5_ gasses in addition to methane (e.g., Mediterranean Amon mud volcano, Mastalerz et al., 2009) and hydrocarbon seeps emit a broad range of alkanes, alkenes and aromatics (e.g., at Guaymas Basin in the Gulf of California, Byrne and Emery, 1960; Simoneit and Lonsdale, 1982). Many microorganisms are able to utilize these hydrocarbons as their carbon source (Widdel et al., 2010). In seep sediments, degradation mainly takes place under anoxic conditions as oxygen is generally depleted within the first few millimeters of the sediment. A large fraction of sulfate reduction (SR) at gas and hydrocarbon seeps is fueled by the anaerobic oxidation of methane (AOM, Reeburgh, 2007). However, as indicated by a global median ratio of SR to AOM of 10.7, a major part of total SR is fueled by the oxidation of non-methane hydrocarbons, in particular the oxidation of alkanes (Bowles et al., 2011).

**FIGURE 1:**
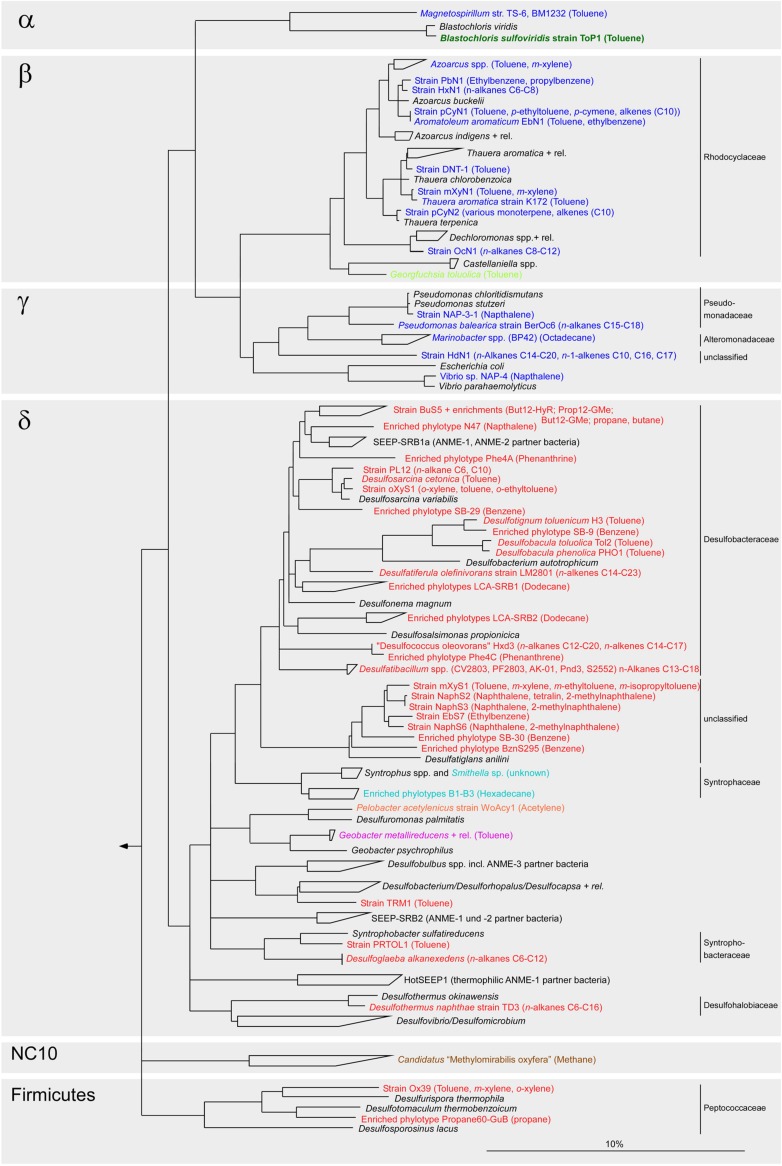
**Phylogenetic tree showing the affiliation of 16S rRNA gene sequences from isolated or enriched anaerobic hydrocarbon degraders to selected reference sequences of the domain *Bacteria*.** Nitrate-reducing bacteria are printed in blue, sulfate-reducing bacteria in red, iron-reducing bacteria in purple, phototrophic bacteria in green, fermentative bacteria in orange and syntrophic bacteria degrading hydrocarbons in a consortium under methanogenic conditions in light blue. *Georgfuchsia toluolica*, printed in light green, has been shown to use Fe(III), Mn(IV), and nitrate as terminal electron acceptor for growth on aromatic compounds. *Cd*. Methylomirabilis oxyfera, printed in brown, can oxidize methane anaerobically by utilizing oxygen produced internally from dismutation of nitric oxide into nitrogen and oxygen gas. Substrate usage is given within parenthesis. The bar represents 10% estimated sequence divergence.

Microbial anaerobic oxidation of alkanes has been described for a large range of alkanes: gaseous (C_2_–C_5_) alkanes (Kniemeyer et al., 2007; Savage et al., 2010; Jaekel et al., 2012; Adams et al., 2013; Bose et al., 2013), mid-chain (C_6_–C_12_) alkanes (Ehrenreich et al., 2000; Davidova et al., 2006) and long-chain (C_13_–C_20_) alkanes (Aeckersberg et al., 1991; So and Young, 1999; Zengler et al., 1999; Cravo-Laureau et al., 2004a). Cultivated anaerobic alkane degraders use sulfate (sulfate-reducing bacteria, SRB), nitrate, manganese or ferric iron Fe(III) as electron acceptors (Weelink et al., 2009; Widdel et al., 2010). Furthermore, hydrocarbon-degrading enrichment cultures have been established under methanogenic conditions (Zengler et al., 1999; Chang et al., 2006; Berdugo-Clavijo and Gieg, 2014; Embree et al., 2014). Isolated or enriched anaerobic alkane degraders belong to two phyla: Proteobacteria and Firmicutes (**Figure 1**). Only recently an archaeon, *Archaeoglobus fulgidus* (Euryarchaeota), has been shown to degrade long-chain alkanes (C_10_–C_21_) with thiosulfate or sulfate (Khelifi et al., 2014). In marine environments alkane degradation is predominantly performed by SRB within the class Deltaproteobacteria and in particular with members of the family *Desulfobacteraceae* (**Figure 1**). Members of the *Desulfosarcina/Desulfococcus* (DSS) clade have been shown to be key players in seep sediments (Knittel et al., 2003; Acosta-González et al., 2013; Kleindienst et al., 2014). Stable isotope probing identified four specialized DSS clades that are active in the oxidation of short- and long-chain alkanes (Kleindienst et al., 2014). Cultivation-independent studies using clone libraries or next generation sequencing techniques (Teske et al., 2002; Orcutt et al., 2010; Ruff et al., 2015) and alkane incubation studies (Savage et al., 2010; Adams et al., 2013; Bose et al., 2013) showed a high diversity of SRB at hydrocarbon seeps suggesting potential for a much larger diversity of alkane degraders as is currently known from available isolates.

Several biochemical reactions have been described for alkane activation under anoxic conditions (Callaghan, 2013; Musat, 2015). The most well-described and particularly dominant pathway is the addition of alkanes to fumarate yielding alkylsuccinates. The enzyme involved in this initial activation step is a glycyl radical enzyme of the pyruvate formate lyase family, the (1-methylalkyl)succinate synthase, Mas (Grundmann et al., 2008), which has also been referred to as alkylsuccinate synthase, Ass (Callaghan et al., 2010). The putative catalytic subunit of Mas is subunit D (MasD), which is equivalent to Ass subunit A (AssA). Alkane activation by MasD has been described for a range of *n*-alkanes and cycloalkanes in sulfate-reducing and nitrate-reducing isolates and enrichment cultures (Kropp et al., 2000; Callaghan et al., 2006; Kniemeyer et al., 2007; Grundmann et al., 2008; Musat et al., 2010; Jaekel et al., 2015). Furthermore, fumarate addition has also been suggested for methanogenic alkane degradation based on the detection of *assA/masD* genes in enrichments (Davidova et al., 2011; Mbadinga et al., 2011; Zhou et al., 2012; Aitken et al., 2013; Cheng et al., 2013). Recently, an *ass*ABC operon was detected on a *Smithella* single cell genome (Tan et al., 2014). Considering the wide distribution of alkane activation via fumarate addition, MasD/AssA serves as valid biomarker for anaerobic alkane degradation. For alkane degradation by achaeon *Archaeoglobus fulgidus*, an alkylsuccinate synthase activity was hypothesized for pyruvate formate lyase (Pfl) D based on a higher similarity of PflD with AssA compared with reference Pfls (Khelifi et al., 2014). Yet, there are only few environmental studies on AssA/MasD diversity in contaminated soils and groundwater (Wang et al., 2012; Zhou et al., 2012; von Netzer et al., 2013), contaminated river sediments and aquifers (Callaghan et al., 2010) or marine hydrocarbon-impacted sediments (Acosta-González et al., 2013; von Netzer et al., 2013; Johnson et al., 2015). Considering the high number of long branches in AssA/MasD phylogenetic trees these studies point to the existence of a broad diversity of microorganisms involved in the degradation of alkanes.

In this study we addressed the diversity of the alkane-degrading microbial community by massive parallel 454-tag sequencing of *masD* genes retrieved from 12 globally distributed marine seep sediments and correlate their characteristics with environmental parameters like hydrocarbon composition, water depth, temperature and SR rates. We hypothesize that in marine seep sediments the diversity of the anaerobic alkane-degrading microbial community is much higher than previously known, including new deeply branching taxonomic lineages. Due to the narrow range of substrate used by isolated alkane degraders (Musat, 2015) we hypothesize that the MasD-microbial community at methane and gas seeps differ significantly from that at hydrocarbon seeps. As an integral part of the study, we optimized the geneFISH protocol to identify alkane-degrading communities *in situ.*

## Materials and Methods

### Sampling Sites

**FIGURE 2:**
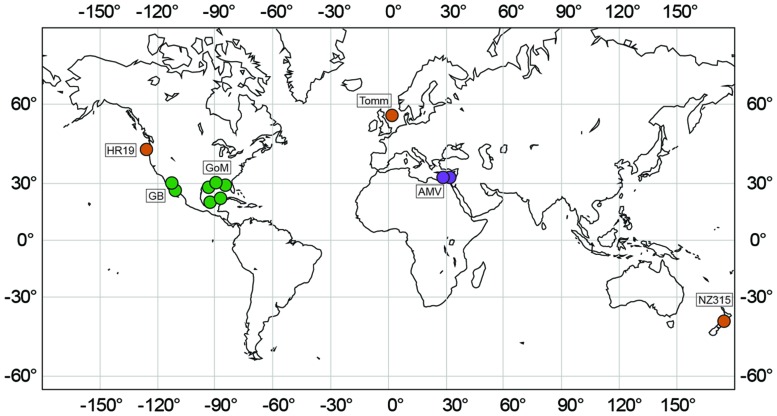
**Marine seep sites investigated in this study.** Green: hydrocarbon seeps, orange: methane seeps; purple: gas seeps.

Sediments were sampled from seven globally distributed marine seeps differing in their geographical, biological, chemical, and geological features (**Table 1**, **Figure 2**). Investigated methane seeps predominantly releasing methane were located on the Cascadian Margin at Hydrate Ridge (Pacific Ocean, station HR19), on the Hikurangi continental margin (New Zealand, Wairarapa, station NZ315) and in the central North Sea (Tommeliten, station Tomm). Gas seeps at the Mediterranean Amon mud volcano (AMV, stations AMV760, AMV825) release significant amounts of other gasses (C_2_–C_5_ alkanes) in addition to methane. Hydrocarbon seeps, in the northern (stations GoM4463 and GoM156), and southern Gulf of Mexico (Chapopote Asphalt Volcano: stations GoM140, GoM13, GoM17) and in the Guaymas Basin (stations GB4573 and GB4484) in the Gulf of California are characterized by seepage of complex hydrocarbons (**Table 1**).

**Table 1:** Description of sampling sites.

Seep type	Sampling site	Station	Cruise; year	Latitude	Longitude	Water depth [m]	Hydrocarbon composition	Reference
Hydrocarbon seeps	Chapopote Asphalt Volcano (Campeche Knolls, Southern Gulf of Mexico)	GoM140	SO174; 2003	21°54.00‘N	93°26.40′W	2902	Mainly methane and C_2_–C_4_ alkanes, few C_29_–C_32_ alkanes	Orcutt et al., 2010; Wegener, unpublished


		GoM13	M67/2; 2003	21°53.99N	93°26.18′W	2908	Mainly steranes, hopanes, few paraffins, alkylbenzenes, cycloalkanes	
		GoM17	M67/2; 2003	21°53.94′N	93°26.14′W	2908	Mainly asphaltenes (>C_20_)	
	Northern Gulf of Mexico	GoM4463	LExEN; 2002	27°44.48′N	91°19.04′W	504	Aromatic hydrocarbons and methane	Orcutt et al., 2010
		GoM156	SO174; 2003	27°46.95′N	91°30.47′W	550	Methane, ethane, C_16_ alkanes, isoprenoids, naphthalene, toluene	
	Guaymas Basin (Gulf of California)	GB4573	AT15-56; 2009	27°00.69′N	111°24.26′W	2100	C_12_–C_38_ alkanes, cycloalkanes, diverse aromatics	Bazylinski et al., 1988
		GB4484	AT15-56; 2009	27°00.64′N	111°40.96′W	2000	C_12_–C_38_ alkanes, cycloalkanes, diverse aromatics	

Gas seeps	Amon Mud Volcano (Mediterranean Sea)	AMV760	M70/2; 2006	32°22.129′N	31°43E	1122	Mainly methane and C_2_–C_4_ alkanes	Mastalerz et al., 2009; Grünke et al., 2011



		AMV825	M70/2; 2006	32°22.128′N	31°42E	1122	Mainly methane and C_2_–C_4_ alkanes	
Methane seeps	Hydrate Ridge (Cascadia Margin)	HR19	SO148-1; 2000	44°34.18′N	125°08.80′W	777	Mainly methane (>95%), a few other gaseous alkanes	Boetius et al., 2000 Treude et al., 2003
	Wairarapa (New Zealand)	NZ315	SO191-3; 2007	41°46.28′S	175°25.78′E	1058	Mainly methane (>99%), a few other gaseous alkanes	Ruff et al., 2013
	Tommeliten (North Sea)	Tomm	AL267; 2004	56°29.90′N	02°59.80′E	75	Mainly methane (>99%), a few other gaseous alkanes	Wegener et al., 2008


### Nucleic Acid Extraction

DNA was extracted by mechanical, chemical and enzymatic cell lysis steps according to the protocol of Zhou et al. (1996) from sediments frozen immediately after sampling at -20°C. Extracted nucleic acids were washed with ice-cold ethanol (80% v/v), centrifuged at 14,000 × *g* for 10 min at RT, dried for 15–30 min at RT and gently resuspended without pipetting in TE buffer (10 mM Tris-HCl, pH 8.0, 1 mM EDTA, pH 8.0) for 1 h at 4°C.

### MasD Amplification and Pyrosequencing

The *masD/assA* gene was amplified using one of two primer pairs: 7757f-1,f-2 (TCG GAC GCG TGC AAC GMY CTG A; MasD amino acid position 395 in strain HxN1; accession number CAO03074)/8543r (TCG TCR TTG CCC CAY TTN GG; position 657 in HxN1) or primer pair 7766f (TGT AAC GGC ATG ACC ATT GCG CT; position 398 in HxN1)/8543r (TCG TCR TTG CCC CAY TTN GG) (von Netzer et al., 2013). Primers were barcoded and extended with an *SfiI* restriction site at the 5′ end for ligation with the 454-adapters. For each sample, eight replicate PCRs (20 μl volume) per primer pair were carried out containing, 0.5 μM primer each, 250 μM dNTPs, 0.3 μg μl^-1^ BSA, 1 × PCR buffer, 0.25 U Taq polymerase (5Prime, Germany) under the following conditions: initial denaturation at 95°C for 5 min, followed by 35 cycles of denaturation (96°C, 1 min), annealing (58°C, 1 min), elongation (72°C, 2 min), and a final elongation step (72°C, 10 min). Replicate PCR reactions of both primer pairs were pooled and the 800 bp-amplicons were then extracted from an agarose gel (1.5% w/v) and purified using the MiniElute PCR Purification Kit (Qiagen) according to the manufacturer’s recommendations. Massive parallel tag sequencing of the amplicons was carried out on a 454 Life Sciences GS FLX sequencer (Roche, Basel, Switzerland) at the Max Planck-Genome-Center, Cologne, Germany.

For pairwise comparison of 16S rRNA genes and MasD (see below) we amplified and Sanger sequenced *masD* from the following alkane-degrading strains and enrichments (**Table 2**): PF2803 (DSM16219, Cravo-Laureau et al., 2004b), LM2801 (DSM18843, Cravo-Laureau et al., 2007), and Propane60-GuB (Kniemeyer et al., 2007).

**Table 2:** Alkane-degrading strains used for calculation of MasD OTU thresholds.

Strain/enrichment	e^-^-acceptor	Phylogenetic affiliation	MasD	16S rRNA
Strain HxN1	NO_3_^-^	Betaproteobacteria	CAO03074	AF331975
Strain OcN1	NO_3_^-^	Betaproteobacteria	CBK27727	AF331976
Strain HdN1	NO_3_^-^	Gammaproteobacteria	NC_014366	AF331974
Strain BuS5	SO_4_^2-^	Deltaproteobacteria	AXAM00000002	EF077225
Propane60GuB	SO_4_^2-^	Firmicutes	LN879422	EF077227
Butane12-Gme	SO_4_^2-^	Deltaproteobacteria	unpublished^a^	EF077226
Strain PL12	SO_4_^2-^	Deltaproteobacteria	LC102219	AB468588
*Desulfoglaeba alkanexedens* (str. ALDC)	SO_4_^2-^	Deltaproteobacteria	GU453656	DQ303457
Strain TD3	SO_4_^2-^	Deltaproteobacteria	Unpublished^b^	X80922
Strain Hxd3	SO_4_^2-^	Deltaproteobacteria	CP000859.1	AF141881
*Desulfatibacillum alkenivorans* (str. AK-01)	SO_4_^2-^	Deltaproteobacteria	CP001322.1	NR_074962
*Desulfatibacillum alkenivorans* (str. PF2803)	SO_4_^2-^	Deltaproteobacteria	LN879420	NR_025795
*Desulfatibacillum aliphaticivorans* (str. CV2803)	SO_4_^2-^	Deltaproteobacteria	AUCT01000049	AY184360
*Desulfatiferula olefinivorans* (str. LM2801)	SO_4_^2-^	Deltaproteobacteria	LN879421	DQ826724
Strain PnD3	SO_4_^2-^	Deltaproteobacteria	Unpublished^b^	Y17501


*^a^Musat and Jaekel, unpublished data.*

*^b^Kirsten Webner: “Die Gene der (1-Methylalkyl)succinat-Synthase im anaeroben n-Alkanabbau des Betaproteobakteriums Stamm HxN1”; Ph.D. Thesis, University Bremen (Germany), 2012.*

### Sequence Processing

Raw reads were submitted to a rigorous quality control using a mothur version 1.29.1 routine (Schloss et al., 2009) including stringent quality filtering and chimera check using UCHIME (Edgar et al., 2011). Sequences were removed from the data set if they had ≥1 mismatch to the forward primer, were <200 bp in length, had >8 homopolymers, had an average quality score <20 (qthreshold = 20) or contained any ambiguities. Sequences were then translated *in silico* (RevTrans 1.4 Server) and screened for MasD based on the presence of the amino acid motive FECIR, FECIK, FECQR, FECVR, FDCIR or FDNIA. Ninety percent of MasD/AssA proteins in our database possess one of these motives at position 435 (HxN1). Sequences with stop codons were removed from the dataset. Sequences were further checked for the presence of the catalytically active cysteine (Grundmann et al., 2008) at position 477 of HxN1. We could not confirm the presence of the conserved motif (RVXG) that harbors the radical-storing glycine (Becker et al., 1999) and is characteristic for all glycyl radical enzymes including MasD and BssA as it lies outside the region amplified by our primers (position 811; HxN1).

### Establishment of a Protein Database for Glycyl Radical Enzymes

We built a comprehensive protein database containing more than 10,000 sequences for MasD, BssA, NmsA and pyruvate formate lyase (Pfl). Sequences were retrieved from this study or from public databases GenBank, NCBI and DDBJ or from publically available complete or draft genomes, single cell genomes, and metagenomes accessible via the Integrated Microbial Genome (IMG) with microbiome samples (IMG/M) system at JGI^1^ (Markowitz et al., 2014). The *mas* operon sequence from strain HxN1 (AM748709) was used for a BlastX search versus the IMG/M database resulting in >1400 potential glycyl radical enzyme sequences. We then imported all amino acid sequences retrieved from these databases along with our own sequences retrieved from seep sediments into the software package ARB (Ludwig et al., 2004) and aligned them using MAFFT v7 (Katoh and Standley, 2013). Manual correction of frame shifts based on BLASTX was necessary for about 15 of our seep sequences due to insertions or deletions caused by 454-pyrosequencing. The amino acid alignment is provided in Supplementary information (**Supplementary Table S1**).

### Definition of OTU Cut-Offs for MasD Amino Acid Sequences

Taxonomic units for MasD were defined based on 15 alkane-degrading pure cultures by pairwise comparison of their amino acid sequences with the corresponding 16S rRNA gene sequences. A list of strains used for this analysis is provided in **Table 2**. The sequence difference D and similarities S (*S* = 1 – D) were calculated. Finally, the similarity of MasD amino acid sequence pairs were plotted versus the similarity of the 16S rRNA gene sequence pairs of the same strains.

### Phylogenetic Tree Reconstruction

The phylogenetic tree based on small subunit ribosomal RNA genes was calculated with nearly full-length sequences (>1350 bp) available in Arb Silva database release 111 (Pruesse et al., 2007^2^) by neighbor-joining analysis in combination with filters which consider only 50% conserved regions of the 16S rRNA. Partial sequences were subsequently inserted into the reconstructed consensus tree by parsimony criteria, without allowing changes in the overall tree topology. MasD-based phylogenetic tree was constructed by maximum likelihood analysis (PhyML algorithm, Blosum 62 substitution model) considering 95 amino acid positions (position 436 to 506, strain HxN1; CAO03074) using 441 deduced amino acid sequences. Only one representative sequence per MasD family level OTU_0.72_ is shown in the final tree.

### Community Diversity Analysis

Sequence abundance tables were generated by clustering the retrieved MasD seep sequences at 96% amino acid identity based on 120 amino acid positions (Pos. 398-500, HxN1) using a distance matrix in Mothur (Schloss et al., 2009) and used to calculate inverse Simpson diversity indices and species rarefaction. Bray–Curtis dissimilarities (Bray and Curtis, 1957) between all samples were calculated and used for two-dimensional non-metric multidimensional scaling (NMDS) ordinations with 20 random starts (Kruskal, 1964). Stress values below 0.2 indicated that the multidimensional dataset was well represented by the 2D ordination. Hierarchical clustering of all samples was performed using Ward’s method (Ward, 1963), which minimizes the total within-cluster variance. A network was built based on a presence absence matrix. The network vertices (nodes) were plotted using a Fruchterman and Reingold (1991) force-directed algorithm, which causes an increase in the nodes attraction to each other with increasing similarity between them; the more OTU_0.96_ shared between two samples, the closer they are in the network. Analyses were carried out with the R statistical environment and the packages vegan (Oksanen et al., 2012), ggplot2 (Wickham, 2009^3^) and network (Butts, 2008) in addition to custom R scripts. *In silico* coverage (C) of MasD sequences was calculated per station according to the following equation

$$C={\left[1-\left(n/N\right)\right]}^{*}100(Good,\;1953),$$

where n is the number of singletons (SSO_abs_ + SSO_rel._) and N the total number of sequences analyzed.

### Design of Probes for *masD* Detection

To set-up a *masD* geneFISH assay, an enrichment culture with *n*-butane was established under sulfate-reducing conditions from hydrocarbon seep sediments of site GB4573. 5 ml sediment slurry was made using a 1:1 mix of sediment from the upper 10 cm with artificial anoxic seawater (Widdel and Bak, 1992) and incubated in hungate tubes containing 5 ml anoxic media for anaerobic sulfate-reducing microorganisms (Widdel and Pfennig, 1982). As substrate, 1 bar *n*-butane gas (Messer Griesheim GmbH, Krefeld, Germany) was added to the headspace. All tubes were kept under N_2_/CO_2_ (90/10, v/v) atmosphere and were horizontally incubated at 28°C. When sulfide production reached 15 mM, 10% of the enrichment culture was subsequently transferred as inoculum into fresh *n*-butane supplemented media resulting in sediment-free enrichment cultures. DNA was extracted from the enrichment GB4573_14 and *masD* genes were amplified using primers *ass/bss* F and *ass/bss* R as described previously (Callaghan et al., 2010). Cloning and sequencing of 689 bp-amplicons was performed as described previously (Kleindienst et al., 2012). Based on 28 sequenced clones used to represent the breadth of diversity of *masD* in our sample, we designed a probe mix consisting of 9 *masD-*targeting dsDNA polynucleotide probes (316 bp; GC content 65%) using the PolyPro software (Moraru et al., 2010, 2011, **Supplementary Table S2**). The individual probes showed >75% nucleotide sequence identity to the target regions of all members of the *masD* gene clusters retrieved from the GB4573_14 enrichment and >72% nucleotide sequence identity to all retrieved MasD OTUs_0.72_. For probe synthesis, plasmid DNA was extracted from 9 selected clones using the SpinMiniprep Kit (Qiagen, Hilden, Germany) and dsDNA probes were synthesized by PCR with enzymatic incorporation of Dig-labeled nucleotides (dUTP) using the PCR Dig Probe Synthesis Kit (Roche, Diagnostics, Mannheim, Germany). As negative control, probe NonPolyPr350 was synthesized. Synthesized dsDNA probes were purified with the GeneClean Turbo kit (Q-Biogene).

### Fluorescence *In Situ* Hybridization of *masD* (geneFISH)

One ml was subsampled from the enrichment culture GB4573_14 at three different time points, fixed with formaldehyde (2% final concentration in 1x PBS (pH 7.4) for 1 h at RT) and an aliquot was filtered on polycarbonate filters (GTTP, pore size: 0.2 μm). Filters were incubated in 0.01 M HCl for 10 min at room temperature to inactivate endogenous peroxidases, followed by incubation in 10 mg/ml lysozyme for 1 h at 37°C to permeabilize cell walls. CARD-FISH targeting 16S rRNA with probe DSS658 (Manz et al., 1998) specific for the deltaproteobacterial clade of *Desulfosarcina/Desulfococcus* was performed as described earlier (Ishii et al., 2004) using Alexa488-labeled tyramide for signal amplification. Following hybridization of 16S rRNA, inactivation of the probe-coupled horseradish peroxidase enzymes (HRP) was achieved by incubations of the filters in 3% H_2_O_2_ in 1xPBS for 30 min at RT followed by incubation in 0.1 M HCl for 10 min. RNAs in the cells were digested by incubating the filters in RNase solution (0.5 U μl^-1^ RNase I, Ambion), 30 μg ml^-1^ RNase A (Sigma), 0.1 M Tris-HCl pH 8 for 4 – 5 h at 37°C. Filter sections, with either the *masD* probe mix or negative control probe NonPolyPr350 were incubated in hybridization buffer containing 45% formamide as calculated by the PolyPro software. After initial denaturation at 85°C for 25 min, hybridization lasted for 18 – 22 h at 50°C followed by binding of the anti-Dig HRP-conjugated antibody (Fab fragments) and signal amplification with a Alexa 594-labeled tyramide. Filter sections were embedded in SlowFadeGold antifade reagent (Invitrogen), containing 1 μg ml^-1^ 4′,6-diamidino-2-phenylindole (DAPI). Microscopy was performed on an epifluorescence microscope (Axioplan, Carl Zeiss). To correct for false positive signals, i.e., extracellular signals and non-specific probe binding to particles or filter matrix, we subtracted negative control counts from the total gene counts.

### Nucleotide Sequence Accession Numbers

*MasD* clone sequences from enrichment culture GB4573_14 have been deposited in the EMBL, GenBank and DDBJ nucleotide sequence database under accession numbers LN610408 to LN610424 and HG764719 to HG764728 and MasD sequences from strains PF2803 (DSM 16219), LM2801 (DSM 18843) and the phylotype Propane60GuB under numbers LN879420- LN879422. Raw *masD* pyrotag sequences have been stored in the sequence read archive under SRA bioproject number 278019.

## Results

### Definition of OTU Cut-Offs for masD

**FIGURE 3:**
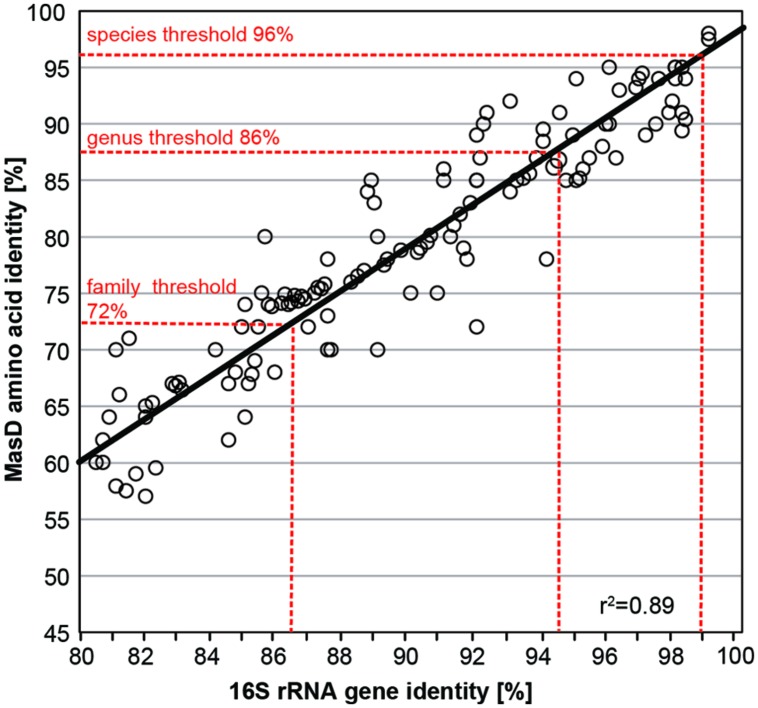
**Pairwise comparison of MasD amino acid and 16S rRNA gene sequence identity for 15 strains capable of anaerobic alkane degradation.** Accession numbers of used strains are given in **Table 2**. The intersection of vertical dashed lines and the regression line gives the cutoff values for defining a species (96%), genus (86%), and family (72%) based on MasD identity corresponding to the proposed values based on 16S rRNA genes (Yarza et al., 2014).

Thresholds for OTU clustering were calculated by linear correlation of 15 cultured alkane-degrading strains with both 16S rRNA genes and MasD sequence information (**Table 2**). The taxonomic threshold for microbial species is 98.7% for 16S rRNA genes (Stackebrandt and Ebers, 2006; Yarza et al., 2014), which corresponded to a threshold of 96% for MasD (OTU_0.96_; **Figure 3**). The 16S rRNA gene genus-level (94.5%) and family level thresholds (86.5%; Yarza et al., 2014) corresponded to cut-off values for MasD of 86% (OTU_0.86_) and 72% (OTU_0.72_), respectively.

### Diversity of MasD Community in Seep Sediments

The diversity of anaerobic alkane-degrading bacteria was studied by pyrosequencing of *masD* present in sediments from 12 globally distributed stations at methane, gas or hydrocarbon seep sites. We retrieved a total of 12,745 raw sequences. Strict quality filtering of raw sequences was an important part of our data analysis as sequencing errors and chimeras affect OTU clustering of protein sequences more severely than clustering of nucleic acid sequences and would result in an overestimation of environmental MasD diversity. After filtering, 40% of raw reads (5,131 *masD* sequences, 652 *nmsA* sequences and 79 *bssA* sequences) were included in further analyses. After translation and clustering the protein sequences on species-level (OTU_0.96_) we obtained 420 MasD OTU_0.96_, 1 NmsA OTU_0.96_, and 1 BssA OTU_0.96_ (**Table 3**). The retrieval of *nmsA* and *bssA* sequences from our seep sites was a result of unspecific binding of our *masD* primers. It is likely then that diversity of *bssA* and *nmsA* is not fully covered by these primers and is omitted from further analysis.

**Table 3:** Mas subunit D (MasD) diversity at investigated hydrocarbon seep sites.

			Observed				Subsampled	
				
Sample	Raw reads	Quality reads	OTU_0.96_ no.	Relative SSO^∗^ [%]	Absolute SSO^∗∗^ [%]	Coverage [%]	OTU_0.96_ no.^∗∗∗^	Inverse Simpson [1/D]
GoM140	535	430	58	36	43	89	17	6
GoM13	751	487	47	47	36	92	16	4
GoM17	892	226	27	63	26	89	15	3
GoM4463	1419	154	44	55	36	74	27	9
GoM156	1078	803	87	32	47	91	21	4
GB4573	1255	989	93	15	49	94	17	7
GB4484	834	572	66	29	55	90	14	4
AMV760	837	134	21	48	43	89	12	3
AMV825	637	376	50	56	32	88	19	3
HR19	708	65	13	62	23	83	13	3
NZ315	2043	722	65	28	52	93	16	3
Tomm	1756	173	33	58	24	84	18	6
Total	12745	5131	420	16	61			
Average							17	5
Median							17	4
*SD*							4	2


*^∗^SSO_*rel.*_, relative single sequence OTU_*0.96*_: sequences occurring only once in at least one sample but may occur more often in other samples, given in percent of OTU _*0.96*_ per station.*

*^∗∗^SSO_*abs.*_, absolute single sequence OTU_*0.96*_: sequences occurring only once in the whole dataset, given in percent of OTU _*0.96*_ per station.*

*^∗∗∗^Standardized numbers of OTU_*0.96*_ based on resampling of 65 sequences without replacement.*

#### MasD Richness and Evenness

Observed richness of alkane degraders ranged between 13 and 93 MasD species-level OTU_0.96_ (**Table 3**). Coverage ranged between 83 and 94% indicating sufficient sampling effort (**Table 3**, **Supplementary Figure S1**) except for the site with the highest inverse Simpson index, GoM4463, for which the coverage was only 74%. OTU abundance for other taxonomic levels is provided in **Supplementary Table S3**. After subsampling to standardize sequencing effort, OTU_0.96_ diversity differed by a maximum factor of 2.25 between sites. Diversity was highest at GoM4463 (27 OTU_0.96_) and GoM156 (21 OTU_0.96_) but clearly lower at GB4484 and all methane seep sites (13–18 OTU_0.96_). Inverse Simpson diversity index (1/D), which takes into account both OTU richness and evenness, ranged between 3 and 9.

### Similarity of MasD Communities at Different Seep Sites

**FIGURE 4:**
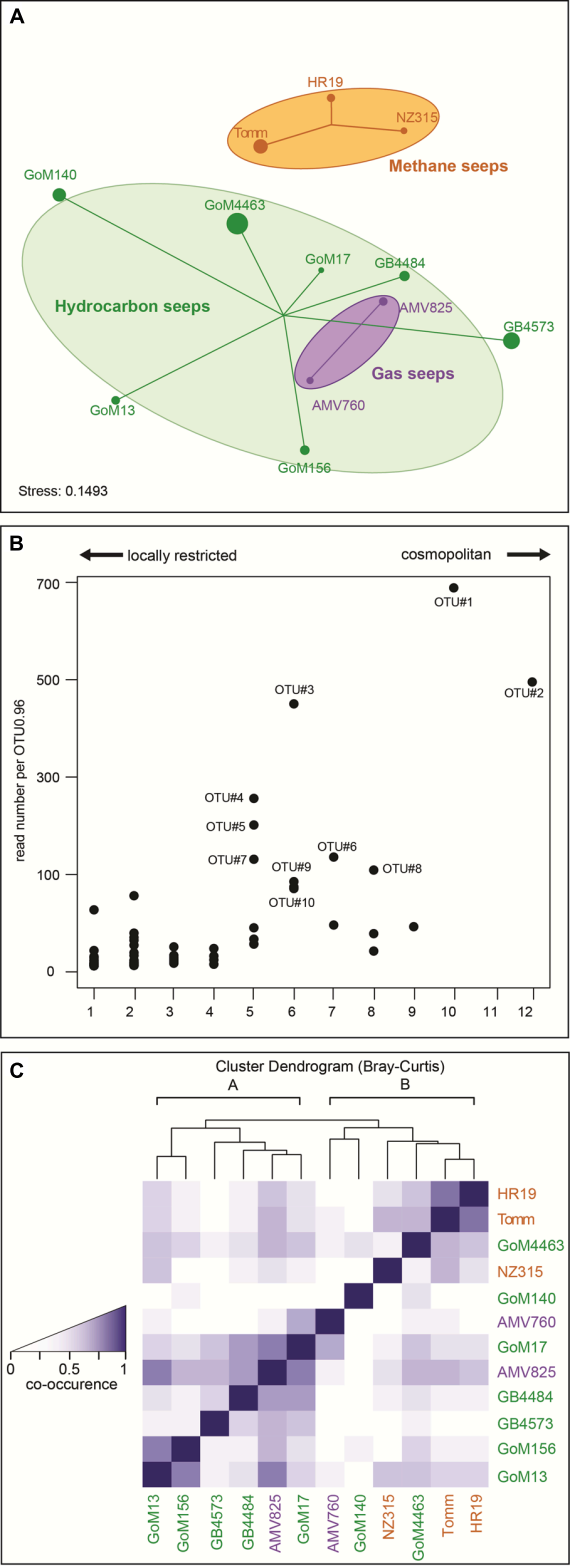
**Similarity of MasD community structure in investigated seep sediments.** **(A)** Similarity of MasD community structure visualized by non-metric multidimensional scaling. Ordination based on species level OTU_0.96_ dissimilarities (Bray–Curtis). Each sample (dot) is connected to the weighted averaged mean of the within group distances. Ellipses represent one standard deviation of the weighted averaged mean. Dot size reflects Inverse Simpson index (1/D), hence the larger a dot the higher the diversity. The mean percentage of shared MasD OTU_0.96_ between any two sites was 9%, lowest was 0% and highest was 43%. **(B)** Abundance and occurrence of MasD OTU_0.96_. Given abundance is based on number of reads per OTU_0.96_. OTU_0.96_ that were present in only 1 of the 12 investigated sediments were defined as locally restricted while those OTU_0.96_ that were present in at least 10 of the 12 sites were defined as cosmopolitan OTU_0.96_. **(C)** Co-occurrence of OTU_0.96_ among the investigated seep sites. The darker the color the more frequent the co-occurrence.

The MasD community structure at different seep sites was visualized by NMDS based on amino acid sequences clustered at species level (OTU_0.96_; **Figure 4A**). Sites were grouped according to hydrocarbon type at the individual sites, i.e., methane (=methane seeps), gaseous hydrocarbons (=gas seeps), or diverse hydrocarbons (=hydrocarbon seeps) and connected to the weighted centroid of the within-group distances. MasD diversity of samples within these three groups showed a high similarity with a high shared proportion of taxa (31%). Dissimilarity was most pronounced between methane seeps and all other sites. Methane seep sites HR19, NZ315 and Tomm revealed quite similar community structures (43% shared taxa) but were clearly different from those at gas and diverse hydrocarbon seeps (<6% shared taxa). AMV gas seeps host MasD communities partially overlapping with those of the hydrocarbon seeps GB and GoM. The frequency of co-occurrence was highest between the hydrocarbon seeps GoM13 and GoM156, between hydrocarbon and gas seeps GB4484, AMV825 and GoM17 and between methane seeps Tomm and HR19.

Hierarchical clustering of co-occurrence frequencies identified two main clusters with cluster A consisting of only gas and hydrocarbon seeps (GoM13, GB4573, GB4484, AMV825, GoM17) and cluster B including all seep types (AMV760, GoM140, NZ315, GoM4463, Tomm, HR19; **Figure 4C**).

### Cosmopolitan and Locally Restricted OTUs

**FIGURE 5:**
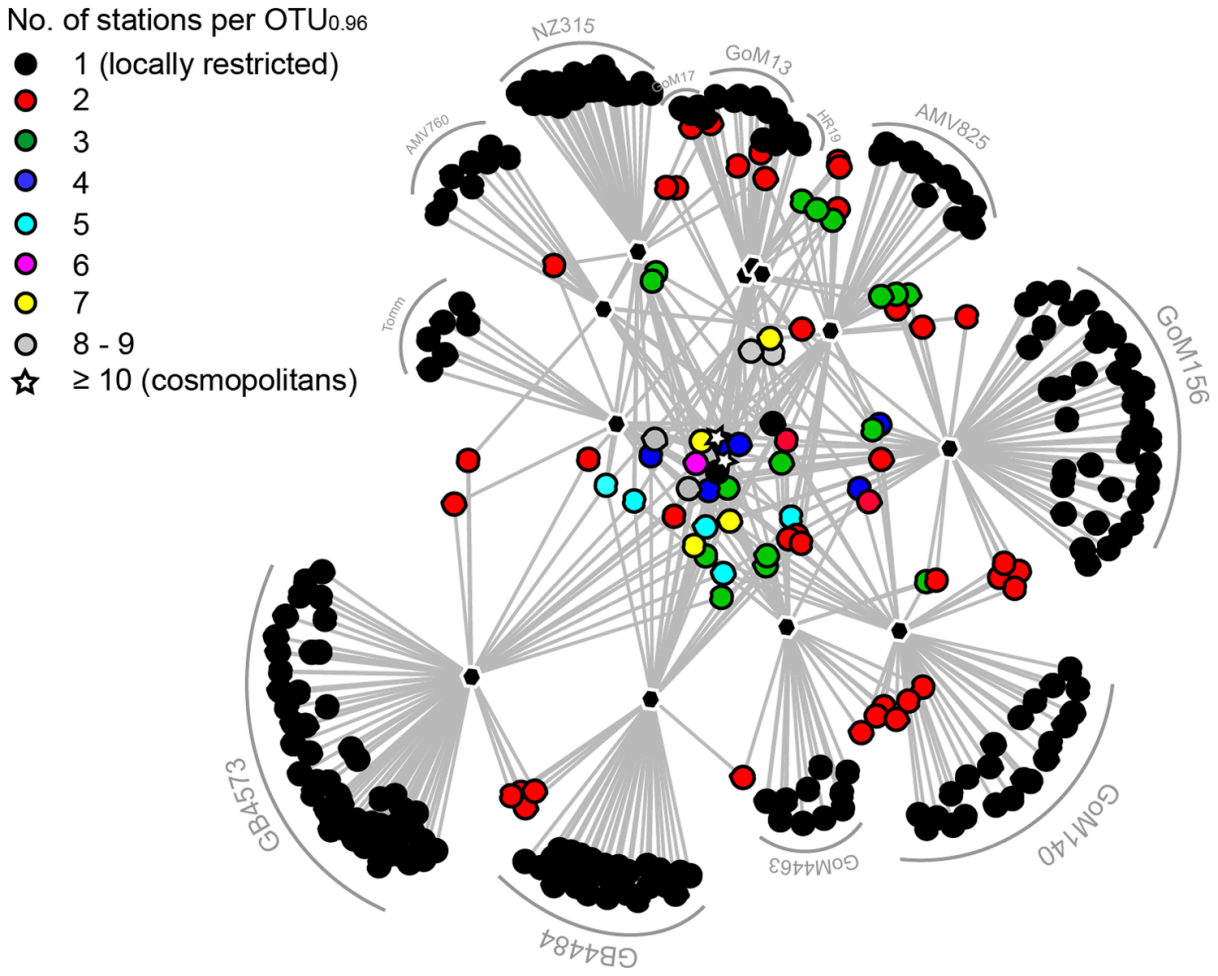
**Network graph displaying the connectivity among sampling sites based on presence-absence of MasD OTU_0.96_.** The different colors indicate the number of investigated seeps that contained an individual OTU. Black dots are locally restricted OTU_0.96_, the stars are cosmopolitan OTU_0.96_. OTU are connected to the sampling sites (black polygons) they occur at.

Two out of the 420 species-level OTU_0.96_ were cosmopolitan as defined by their presence in at least 10 of the 12 stations investigated (**Figures 4B** and **5**). These two OTU comprised the majority of MasD sequences. Fifteen percent of total MasD sequences were assigned to OTU#1 that dominated at almost all sites with 5–55% of quality sequences retrieved from the individual sites. OTU#1 was rare only at methane seep sites NZ315 and Tomm with <2%. Conversely, OTU#2 dominated these two methane seep sites with 53% of total quality reads at NZ315 and 22% at Tomm, in addition to 52% at HR19. The third most abundant OTU#3, which was present at six sites (**Supplementary Table S4**), strongly dominated in GoM13 (44% of the reads), GoM156 (26%) and AMV825 (14%) sediments. In general, the next ten abundant OTU#4 to #13 were only occasionally abundant and found to be dominant at a single seep site (**Supplementary Table S4**). For example, OTU#11 comprised only 2.8% of total sequences and were found at five sites but dominated GoM17 (22%) and AMV760 (65%).

Rare OTU_0.96_ appearing only once in the whole data set (i.e., absolute single sequence OTUs; SSO_abs_) are referred to as ‘locally restricted.’ A majority of OTU_0.96_ (61%) were assigned to SSO_abs_. Conditionally rare organisms are those appearing only once in a given sample but more often in one or more of the other samples (i.e., relative single sequence OTU_0.96_, SSOrel, Gobet et al., 2012). The conditionally rare microbial MasD community comprised 16% of total OTU_0.96_ of which >50% were represented by a low read number (<10 reads per OTU).

### Phylogenetic Affiliation of MasD

**FIGURE 6:**
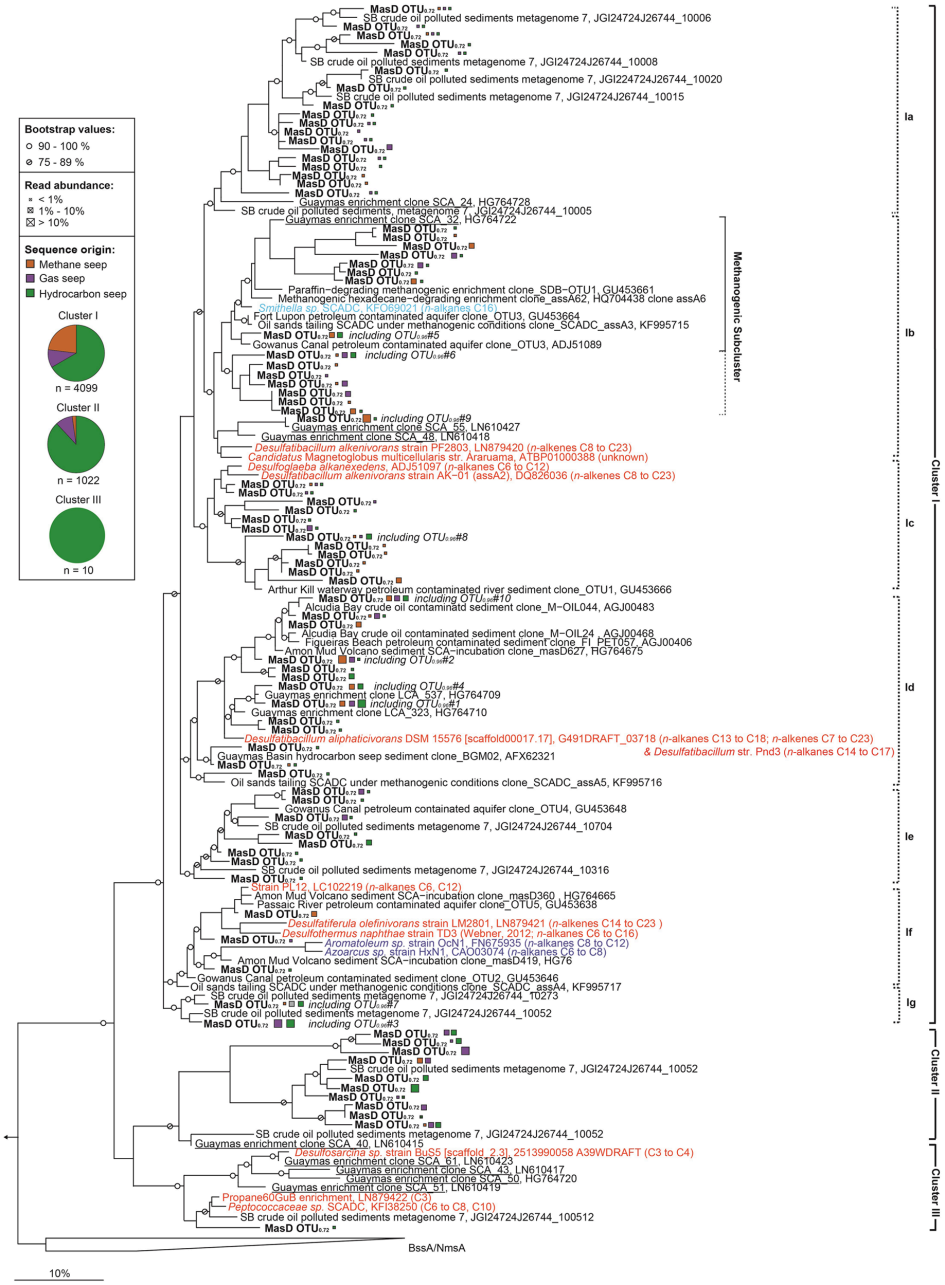
**Phylogenetic tree showing the affiliations of MasD amino acid sequences retrieved from seep sediments with selected reference sequences.** The phylogenetic tree was calculated using the maximum likelihood algorithm (PhyML) with 1000 bootstrap replicates and blosum62 correction considering 95 amino acid positions. The tree was rooted using pyruvate formate lyase (Pfl) as outgroup. Sequences from cultivated alkane degraders or metagenomic analysis were included as additional reference sequences; substrate usage is given within parenthesis. Strains PF2803, LM2801 and enriched phylotype Propane60GuB were added to the tree using parsimony criteria. Nitrate-reducing bacteria are in blue, sulfate-reducing bacteria in red and syntrophic bacteria degrading hydrocarbons under methanogenic conditions in light blue. Sequences from this study are in boldface type. Only one representative amino acid sequence of the individual family level OTUs_0.72_ is shown. MasD sequences for geneFISH probe design, which were retrieved from a clone library constructed from Guaymas Basin enrichment with butane, are underlined. The scale bar gives 10% estimated sequence divergence. Pie charts represent the portion of sequences from a certain seep type assigned to the cluster. Orange = methane seep, purple = gas seep, green = hydrocarbon seep. Abbreviations: Ass, alkylsuccinate synthase, Mas, 1-methyl alkyl succinate synthase, Bss, benzylsuccinate synthase, Nms, naphthyl-2-methyl-succinate synthase.

For phylogenetic analysis, MasD was clustered on a proposed family level of 72% similarity (OTU_0.72_). In total, 83 family level OTUs were detected. Representative sequences formed three clusters that shared <60% sequence similarity (**Figure 6**). The three clusters might comprise organisms from different phyla because the inter-cluster identity of <60% is close to the estimated MasD phylum level OTU threshold at 52% (according to the phylum threshold of 75% for 16S rRNA genes; Yarza et al., 2014). The proposed three phyla are mostly consistent with the 16S rRNA taxonomy of the cultured organisms. Strains within cluster I all belong to Proteobacteria, cluster II does not contain any sequences from cultured strains and cluster III includes sequences from Proteobacteria (strain BuS5, Deltaproteobacteria) but also from Firmicutes.

OTU richness was highest in cluster I: 372 species-level OTU (OTU_0.96_) were identified comprising 4099 sequences (80% of total) with 72 total family level OTU (OTU_0.72_). In cluster II, 47 species-level OTU_0.96_ were identified comprising 1022 sequences (20% of total) with 10 family level OTU_0.72_. And finally cluster III contained only 10 sequences clustered into a single OTU on species, genus and family level (**Figure 6**). For cluster I we defined seven monophyletic subclades: cluster Ia to Ig. Clusters Ia, Ie, and Ig only contain MasD from uncultivated organisms. Clusters Ia and Ib are comprised of a particularly high number of family level OTU_0.72_ (18 and 16, respectively). Cluster Ib also included the AssA found in a draft genome from *Smithella* SCADC, a syntrophic deltaproteobacterium derived from different methanogenic alkane-degrading enrichment cultures (Embree et al., 2014; Tan et al., 2014), and *Candidatus* Magnetoglobus multicellularis (ATBP010000388 on IMG/M). The only cultivated relative in cluster Id is the long-chain alkane- and alkene-degrading *Desulfatibacillum aliphaticivorans*. This cluster includes the two most abundant and cosmopolitan OTU_0.96_ #1 and #2. Cluster If is quite diverse and comprised of MasD from betaproteobacterial nitrate reducers and deltaproteobacterial sulfate reducers but also from medium- or short-chain alkane degraders (**Figure 6**).

### *In Situ* Identification of Alkane-Degrading Bacteria by *masD* geneFISH

**FIGURE 7:**
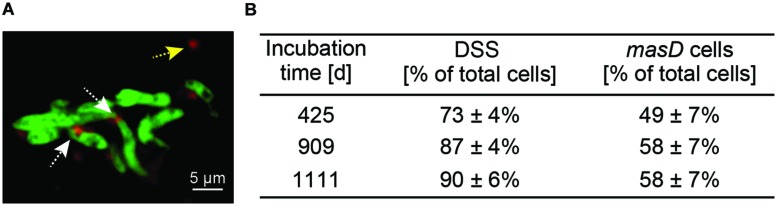
**GeneFISH on alkane-degrading enrichment cultures from Guaymas Basin sediments grown under sulfate-reducing conditions.** **(A)** Fluorescence micrograph showing CARD-FISH stained *Desulfosarcina/Desulfococcus* cells in green (16S rRNA-targeted probe DSS658) and geneFISH-stained *masD*-carrying cells in red (*masD* probe mix). White arrows indicate DSS cells with *masD*-geneFISH signals; yellow arrow indicates non-specific signal. Scale bar = 5 μm. **(B)** Relative abundance of DSS and *masD*-carrying cells in enrichments.

A geneFISH assay for *masD* was established to identify the alkane-degrading bacterial community in the environment. A sediment-free culture from GB4573 sediments grown under sulfate-reducing conditions with *n*-butane as sole carbon source was used to optimize the protocol. The enrichment is dominated by alkane-degrading SRB of the SCA2 clade belonging to the deltaproteobacterial *Desulfococcus/Desulfosarcina* (DSS) branch (Kleindienst et al., 2014). As a basis for probe design, a *masD* gene library was constructed from this enrichment culture. The obtained sequences fall into all three defined clusters (**Figure 6**). Because similarity between clusters I, II, and III was too low on the DNA level to allow the design of a single dsDNA polynucleotide probe covering the whole diversity of the gene (**Figure 6**, **Supplementary Table S1**), clusters were targeted independently. For cluster II, it was possible to cover all sequences with one dsDNA polynucleotide probe. For cluster I and III, we designed probe mixes of 4 dsDNA polynucleotide probes each for complete coverage. The DNA-based sequence similarity between MasD OTU_0.72_ from the 12 seeps and the individual probes was above the threshold of 72%, which has been reported to be detected by a 350 bp probe (Moraru et al., 2010). The dsDNA *masD* probes were synthesized separately and applied as mix on the GB enrichment culture. Hybridization of the enrichment culture showed *masD* gene signals co-localized with signals of probe DSS658 (**Figure 7A**), which targets the *Desulfosarcina/Desulfococcus* branch of Deltaproteobacteria, indicating a successful *in situ* identification of alkane-degrading bacteria. Members of the DSS made up between 73 and 90% of total DAPI-stained cells. Of these cells, 49–58% showed positive *masD* gene signals with the *masD* probe mix (**Figure 7B**).

## Discussion

### Diversity of Alkane-Degrading Bacteria

To date, only about 20 bacterial strains or phylotypes in enrichments have been found to degrade alkanes anaerobically. They belong to a total of ten families within the phyla Proteobacteria and Firmicutes (**Figure 1**). In contrast, a quite high bacterial 16S rRNA gene diversity has been reported for hydrocarbon seeps in the Guaymas Basin and Gulf of Mexico, in particular for deltaproteobacterial SRB (Teske et al., 2002; Dhillon et al., 2003; Orcutt et al., 2010; Wegener and Knittel, unpublished data), suggesting that there are likely many more SRB and other bacteria than currently known that thrive anaerobically on alkanes. Our data clearly support this hypothesis as we found a total of 420 MasD species-level OTU_0.96_ and 83 family level OTU_0.72_ from the 12 investigated hydrocarbon-impacted sites. Based on rarefaction curves, diversity was not fully covered; therefore increased sequencing efforts might reveal even more diversity. Furthermore, we also might have missed new lineages which did not have the sequence motifs we were searching for. For example, we excluded about 70% of reads retrieved from GoM4463 and HR19 from the analysis only due to the absence of these motifs. The high overall MasD diversity can be explained either by the presence of a microbial seed bank, which comprises dormant alkane-degrading organisms that are resuscitated following environmental changes (Lennon and Jones, 2011) or by the presence of many niches as alkanes are abundant in nature.

The oily sediment site from the northern Gulf of Mexico (GoM4463) had the highest MasD α-diversity (Inverse Simpson Index), followed by site GB4573 in the Guaymas Basin, indicating that these habitats are species-rich because of the presence of very diverse hydrocarbons. This high diversity might also enable the microbes to withstand certain environmental changes, like the strong temperature gradients found at Guaymas Basin. In contrast, the asphaltic sample GoM17 was the least diverse, supporting the notion that in an extreme environment with a rather limited buffet of degradable hydrocarbons, small changes would have serious impact on the microbial communities where few new species accumulate.

### Cosmopolitan and Rare Alkane-Degrading Bacteria

Two abundant cosmopolitan species-level MasD OTU_0.96_ were detected in this study indicating that only a small number of dominant alkane degraders are globally distributed. These OTU were assigned to cluster Id, which included *Desulfatibacillum aliphaticivorans* str. CV2803, a sulfate-reducing long-chain alkane- (C_13_–C_18_) and alkene- (C_7_–C_23_) degrading bacterium isolated from hydrocarbon-polluted sediments in the Gulf of Fos (France, Cravo-Laureau et al., 2004a). Their closest relatives were MasD sequences (e.g., Guaymas clone LCA_537) retrieved from an enrichment with dodecane (Kleindienst et al., 2014) suggesting long chain alkanes as substrates for the organisms representing OTU#1 and #2. Their cosmopolitan presence suggests an as of yet unknown environmental importance of members in this group for the degradation of long-chain alkanes and alkenes in marine seep sediments. Cosmopolitan distribution was also shown for other seep-associated organisms, such as certain ANME that have been found in seep sediments worldwide (Ruff et al., 2015).

A tremendous amount of species-level single sequence OTU_0.96_ (SSO_abs_ and SSO_rel_) was retrieved from the 12 different sediments. SSO_abs_ are locally restricted, permanently rare species (Gobet et al., 2012). In contrast, SSO_rel_ are organisms that are rare in one ecosystem, but very common or even dominant in another. These organisms may start growth when the conditions change, which was shown for pelagic communities where rare organisms became abundant after disturbance (Sjöstedt et al., 2012) or showed seasonal patterns (Hugoni et al., 2013). Guaymas Basin site 4573 had the most SSO_rel_ and SSO_abs_ supporting the uniqueness of this habitat with strong geochemical gradients, in particular temperature gradients, previously reported (Teske et al., 2002; Teske and Sørensen, 2008). These minor MasD variants have developed under specialized circumstances that may be linked to the diversity and the structure of the alkanes in that environment.

### Factors Driving the Alkane-Degrading Community Structure

The MasD community structure was so clearly driven by the hydrocarbon source at the various seeps that the pressure of other factors such as water depth has little to no effect. MasD OTU from methane, gas and hydrocarbon seep sites was most similar within the habitat type as shown by non-metric multidimensional scaling. Thus, the range of available alkanes seems to strongly influence the diversity of MasD-carrying microbes. This is consistent with the narrow substrate range of cultured strains. For example, strain BuS5 oxidizes only propane and butane (Kniemeyer et al., 2007), *Azoarcus* sp. str. HxN1 oxidizes C_6_–C_8_ (Ehrenreich et al., 2000) and *Desulfatibacillum aliphaticivorans* str. CV2803 oxidizes C_13_–C_18_ (Cravo-Laureau et al., 2004a). It has been shown that seep-associated anaerobic methanotrophs (ANME clades) are structured by sediment depth and sediment temperature, (Ruff et al., 2015) while sulfate reducers (SEEP-SRB clades) are structured more by faunal activity and thus biogeochemistry (Felden et al., 2014). In all studies, water depth was not identified as an influencing factor.

### Phylogeny of Anaerobic Alkane-Degrading Bacteria

For 16S rRNA genes there are widely accepted thresholds for the definition of a species, genus or family (Rosselló-Móra and Amann, 2015). With the clustering of protein-coding genes, however, we must consider the high variation in sequence conservation. To establish taxonomic levels based on MasD we used cultivated alkane-degrading species and correlated their 16S rRNA nucleotide sequences with their MasD amino acid sequences. This approach has also been implemented for methane-oxidizing bacteria (PmoA, Degelmann et al., 2010) and nitrogen-fixing bacteria (NifH, Bowen et al., 2013). We determined the following threshold values for MasD: 96% amino acid identity for species, 87% for genus, and 72% for families. The species threshold of 96% that we defined for MasD was higher than those determined for PmoA (93%) and NifH (88%), which can in part be explained by the recent re-evaluation and implementation of a new 16S rRNA gene species threshold of 98.7% (Yarza et al., 2014) used in our study compared to the 97% identity threshold used in the above previous studies.

Phylogenetic analysis of MasD resulted in three clusters of which the first was dominant, the second regularly present and the third very rare. The 60% amino acid identity between these clusters is likely in the range of a phylum level threshold for MasD. The only available non-deltaproteobacterial MasD is from phylotype Propane60GuB (cluster III) and showed a maximum of 56% amino acid identity to any sequence of cluster I and cluster II.

Based on the MasD protein sequences alone we cannot exclude that the respective enzymes have similar substrate spectra or affinities. In the MasD phylogenetic tree (**Figure 6**) there was also no obvious separation between sequences retrieved from freshwater (Callaghan et al., 2010) and those marine habitats (Kleindienst et al., 2012; Acosta-González et al., 2013). Thus, cultivation attempts as well as enzyme characterizations are necessary to evaluate phylum-level functional diversity of MasD.

The dominant cluster I was most diverse including several isolated deltaproteobacterial and betaproteobacterial sulfate- and nitrate-reducing bacteria. It was present at all sites suggesting that these microbes are able to adapt to the local environmental features which differ remarkably between seeps. Within subcluster Ib, eight OTU_0.72_ cluster tightly with MasD from methanogenic enrichments and *Smithella* sp. We hypothesize that these MasD belong to syntrophic methanogenic hydrocarbon-degrading communities that are relevant for alkane degradation at marine seeps. However, so far studies on methanogenic alkane-degradation are limited to enrichment cultures (Zengler et al., 1999; Chang et al., 2006; Berdugo-Clavijo and Gieg, 2014; Embree et al., 2014). *In situ* studies that aim to quantify the responsible syntrophic bacteria and archaea directly in their environment have not been conducted, although the importance of crude oil biodegradation via methanogenesis has been proposed for subsurface oil reservoirs (Jones et al., 2008). The anaerobic biodegradation of octacosane (C_28_), a solid paraffin, under methanogenic conditions (Davidova et al., 2011; Callaghan, 2013), could be one process of interest for future *in situ* studies.

MasD of the only cultured short-chain alkane-degrading strain BuS5 (Musat, 2015) grouped into cluster III. Although this strain is found in numbers of ca. 2% of total cell counts at AMV and Guaymas Basin (Kleindienst and Knittel, unpublished data), we did not retrieve any OTU related to BuS5-MasD. Their absence is most likely due to a substantial primer mispairing; the forward primers had 11 (7757f1-f2, 22mer) and 13 (7766f, 23mer) mismatches, respectively, to the BuS5-*masD* sequence retrieved from the isolate genome (JGI gene ID 2513990058). In addition to deltaproteobacterial BuS5, cluster III is also comprised of two Firmicutes, i.e., a single cell from family Peptococcaceae and a phylotype from the enrichment Propane60GuB, indicative of a lateral gene transfer event. We also report a second potential instance of lateral gene transfer in MasD cluster I, which contains both Beta- and Deltaproteobacteria in subclusters.

Despite the broad diversity of cluster II (10 family level OTU_0.72_ from this study), this cluster is comprised of only two other sequences from a metagenomic study from Santa Barbara oil seep sediments (Hess, JGI metagenome, IMG submission ID 26744). To date few environmental studies have been conducted and most have investigated fresh water environments (Callaghan et al., 2010; Cheng et al., 2013) and few marine environments (Acosta-González et al., 2013; Kimes et al., 2013; von Netzer et al., 2013; Johnson et al., 2015). All of them implement clone libraries with very limited sequencing depth.

Considering the high overall diversity found within MasD, the range of substrates currently known to be activated by MasD/AssA might expand with further studies. For example, ethane is the most abundant short-chain non-methane alkane at several of our sites (Bazylinski et al., 1988; Boetius et al., 2000; Orcutt et al., 2008; Felden et al., 2013). However, isolation of anaerobic ethane-degrading organisms is lacking. Recently, sequences retrieved from a Gulf of Mexico batch reactor with ethane and sulfate have been shown to be closely related to *Desulfosarcina variabilis* and strain BuS5 (Bose et al., 2013). Nevertheless, cultivation or enrichment is necessary in order to get detailed insights into the metabolic pathways and substrate spectrum. Large metagenomic studies with systematic screening for *masD* will help to further unravel MasD diversity and improve current primer sets for future studies. Another approach that could prove useful in future *masD* marker gene surveys is oligotyping, which uses only the most informative nucleotide positions to define phylogenetically distinct oligotypes (Eren et al., 2013).

### *In Situ* Identification of Alkane-Degrading Communities

In this study we also used a comparative sequence analysis of *masD* for the development of a polynucleotide probe set. We demonstrated that these probes could be used for the *in situ* identification of bacteria catalyzing the anaerobic degradation of alkanes by geneFISH. The individual geneFISH probes designed in this study could also be used to target subpopulations, i.e., MasD cluster I, cluster II, and cluster III (**Figure 6**). geneFISH was originally invented for *pmoA* and applied on *Escherichia coli* clones in order to link function with phylogeny (Moraru et al., 2010). Later, the method was applied on thin sections of the hydrothermal vent mussel *Bathymodiolus* to detect uptake hydrogenases *(hupL)* in its symbionts (Petersen et al., 2011). Here, we showed that direct application of geneFISH to sediment samples from Guaymas Basin was also possible but challenging due to high background fluorescence.

The *in situ* quantification of *masD* will help to identify the size of alkane-degrading communities in the environment independent of cultivation and enrichment and independent from *a priori* knowledge regarding which taxa are capable of alkane degradation. This method is and will be a major step forward to begin to describe the global relevance and diversity of alkane degradation at natural hydrocarbon seeps and in oil-polluted environments.

## Conclusion

As alkane is the predominant component of crude oil, our findings point to a so far overlooked high potential of marine benthic microbes to react to natural changes in hydrocarbon seepage or to massive hydrocarbon input as encountered during anthropogenic oil spills. The number of MasD OTU_0.72_ that we detected in this study indicated that there may be eight times more family level bacterial clades that are capable of hydrocarbon degradation than previously assumed. This observed high diversity of MasD might allow for higher rates of survival and adaptability within a microbial community exposed to such unstable and changing environmental conditions. Exploring the prevalence and diversity of MasD can help to identify novel lineages of alkane degraders as well as to differentiate closely related phylotypes. Several MasD clusters were discovered that do not yet have cultured representatives. Therefore, one major objective for future studies should be enrichment and isolation of these lineages for further characterization of substrate specialization. Further methodological approaches could include metagenomics and single cell genomics of FACS sorted cells carrying *masD* genes.

Furthermore, this study represents a first step toward establishing comparative MasD sequencing and *masD* geneFISH as powerful tools for targeted investigation of the diversity and abundance of alkane-degrading bacteria in anoxic environments.

## Conflict of Interest Statement

The authors declare that the research was conducted in the absence of any commercial or financial relationships that could be construed as a potential conflict of interest.

## Footnotes

1 http://img.jgi.doe.gov/m

2 www.arb-silva.de

3 http://ggplot2.org/
