# Supplementary material for: High Diversity of Anaerobic Alkane-Degrading Microbial Communities in Marine Seep Sediments Based on (1-methylalkyl)succinate Synthase Genes
Source: Front Microbiol. 2016 Jan 7;6:1511. doi: 10.3389/fmicb.2015.01511 (PMC4703814; doi:10.3389/fmicb.2015.01511)
Supplement: Supplementary file 1 [file Data_Sheet_1.PDF]

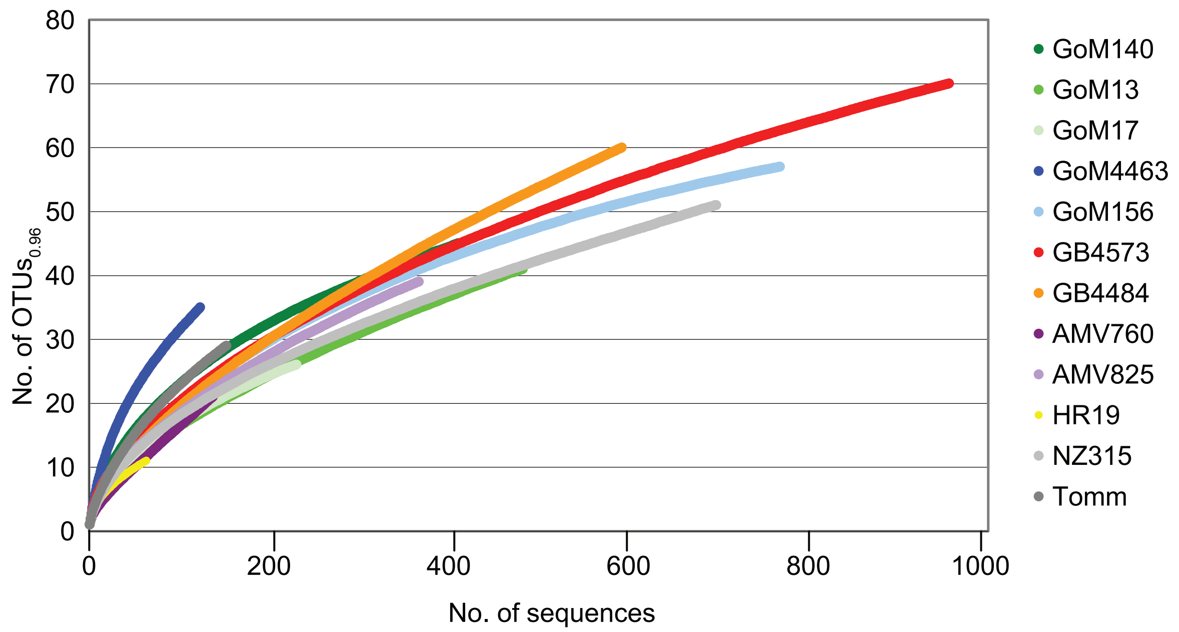

Stagars et al.
Figure S1. Rarefaction curves of MasD OTUs$_{0.96}$ retrieved from 12 marine seep sites

Stagars et al.

Table S1. Partial alignment of MasD amino acid sequences (clades I-III) with sequences from reference glycyl radical enzymes (MasD/AssA, BssA and NmsA) from hydrocarbon-degrading pure cultures. A conserved sequence motif in MasD contains a cysteine (HxN1 positition 477, printed in red) which is assumed to form a thiyl radical by hydrogen atom transfer to the glycyl radical and then to activate the alkane by homolytic C—H bond cleavage (Himo, 2005. Biochim Biophys Acta 1707: 24–33).

Sequence name	Acc. no	Alignment
Azoarcus sp. strain HxN1	CAO03074	FECIRQGLGYPTLRN-DPVLIQNTMHWY-------G-HP-LEEA-RTWVHMACMSPNPTTKHGTSPFRMASATMNS-AKTIEY
Arthur Kill waterway petroleum contaminated river sediment clone_OTU1	GU453666	FECIRHGLGYPTLRN-DPVLVANAMYWH-------G-HP-IEEA-RTWVHQACMSPCPTTKHGTQPMRMAAATANS-AKMIEY
Fort Lupon petroleum contaminated aquifer clone_OTU3	GU453664	FECIRHGLGYPSMRN-DPVLIANSMNWH-------G-HP-IEEA-RTWVHQACMSPCPTTKRGFQPMRMASATANC-AKIMEY
Paraffin-degrading methanogenic enrichment clone_SDB-OTU1	GU453661	FECIRHGLGYPSMRH-DPILIANTMHWY-------G-HP-IEEA-RTWVHQACMSPCPTTKAGFQPMRMANATSNT-AKIIEY
Gowanus Canal petroleum containated aquifer clone_OTU4	GU453648	FECIRHGLGYPSIRH-DPILIANGMYWH-------G-HP-LKEM-RTWVHQACMSPCPXTKHGTQPARMASATLNC-AKMMEY
Gowanus Canal petroleum contaminated sediment clone_OTU2	GU453646	FECIRHGLGYPSMRN-DPVLIENTVYWY-------G-HP-LEEA-RTWVHQACMSPCPTTKHGFQPMRMASATANT-AKMIEY
Passaic River petroleum contaminated aquifer clone_OTU5	GU453638	FECIRHGLGYPSMRN-DPVCIENTVYWY-------G-HP-VEEA-RTWVHQACMSPCPTTKHGFQPMRMASATANC-AKMIEY
Desulfatibacillum alkenivorans strain AK-01 (assA2)	DQ826036	FECIRHGLGYPSFRH-DPILVANCMNWH-------G-HP-VEEA-RTWVHQACMSPCPTTKNGVQPFRMASATANC-AKMVEY
Desulfoglaeba alkanexedens	ADJ51097	FECIRHGLGYPSMRN-DPILVQNAMYWH-------G-HP-LEEA-RTWVHQACMSPCPTTKHGFQPFRMASATANC-AKMIEY
Desulfobacter sp. strain NaphS3	CA072220	FENIAAGFGFPSIKH-EEKNTRQMLEHY-------K-VP-PDEAAH-WALVLCMAPGVSKRRGLQKTRTEGGGLIWIDKCCEI
Aromatoleum sp. strain OcN1	FN675935	FECIRQGLGYPTMRN-DPVLIQNTMHWY-------G-HP-LEEA-RTWLHMACMSPAPTTKHGTSPFRMASATMNS-AKVIEY
Alcudia Bay crude oil contaminated sediment clone_M-OIL24	AGJ00468	FECIRHGLGYPSMRN-DPVLVQNTTHWY-------G-HP-VEEA-RLWVHQACMSPCPPTKHGNQPFRMASATANC-AKIVEY
Figueiras Beach petroleum contaminated sediment clone_FI_PET057	AGJ00406	FECIRHGLGYPAMRN-DPVLIQNSTHWH-------G-HP-VEEA-RLWVHQACMSPCPPTKHGNQPFRMASATANC-AKIVEY
Alcudia Bay crude oil contaminatd sediment clone_M-OIL044	AGJ00483	FECIRQGLGYPSMRN-DPILVQNAMHWH-------G-HP-LEEA-RLWVHQACMSPCPPTKHGYQPFRMASATANC-AKIVEY
Guaymas Basin hydrocarbon seep sediment clone_BGM02	AFX62321	FECIRHGLGYPSMRN-DPVLIANAMHWH-------K-HP-LEEA-RTWVHQACMSPCPTTKHGAQPFRMASATANT-AKIVEY
Amon Mud Volcano sediment SCA-incubation clone_masD360	HG764665	FECIRHGLGYPSMRN-DPVCVENTVYWY-------G-HP-LEEA-RTWVHQACTSPCPTTKHGFQPMRMASATANC-AKMIEY
Amon Mud Volcano sediment SCA-incubation clone_masD419	HG764672	FECIRHGLGYPSMGN-DPILVENTVYWY-------G-HP-LEEA-RTWVHQACMSPCPTTKHGFQPLRMASATANC-AKMVEY
Guaymas enrichment clone SCA_43	LN610417	FECIRHGLGYPNIRN-DEVLIKSQKYWS-------N-YT-EEEA-RTWVAQVCIVPCPESKECVIPARYASCTPLG-SKCLEL
Guaymas enrichment clone SCA_50	HG764720	FECIRHGLGYPNIRN-DQTLIKAAQYWS-------N-YS-EEDA-RAWVAQACIVPCPETKTALIPARLASCTPLG-SKCLEL
Guaymas enrichment clone SCA_51	LN610419	FECIRHGLGYPNIRN-DQVLIKANMFWS-------N-TP-EEEA-RTWTAQACIVPCPETKRGCMPARYSSSATFG-SKSMEL
Guaymas enrichment clone SCA_24	HG764728	FECIRHGLGYPSMRN-DPVLVDNFMYWF-------G-HP-LHEA-RQWVVQACMSPCPPTKQGCQPMRMA-VVCDS-AKIIEY
Guaymas enrichment clone SCA_32	HG764722	FECIRHGVGYPSMGN-DPILVANAMNWH-------G-HP-LEEA-RQWVHQACMSPCPTTKYGCQPMRMASATANC-AKIIEY
Guaymas enrichment clone SCA_48	LN610418	FECIRHGLGYPSMRN-DPILVANAMNWH-------G-HP-IEEA-RTWVHQACMSPCPTTKHGFQSMRMASATANC-AKIIEY
Guaymas enrichment clone SCA_55	LN610427	FECIRHGLGYPNIRH-DPIMVANAMHWH-------G-HP-IEEA-RLWVNQACMSPCPPTKHGQQPMRMASATANC-AKIIEY
Guaymas enrichment clone SCA_61	LN610423	FECIRHGLGYPNIRN-DQVLIKANMFWS-------N-TP-EEEA-RTWVAQACIVPAPETKHGCMPMRYSSCTTLG-SKCMEL
Guaymas enrichment clone SCA_40	LN610415	FECIRHGLGFPQMRH-DEVLIANLMHNF-------G-QP-LRDA-RTWAHMACLSPGPMTKYGGQAIRYSAVSVFA-GKCVEL
Methanogenic hexadecane-degrading enrichment clone_assA62	HQ704438	FECIRHGLGYPSMRN-DPLLIANSMYWH-------G-HP-IEEA-RTWVHQACMSPCPTTKKGSQPMRMANATANC-AKIIEY
Amon Mud Volcano sediment SCA-incubation clone_masD627	HG764675	FECIRHGLGYPSMRN-DPVLVQNAMHWH-------G-HP-LEEA-RLWVHQACMSPCPPTKHGNQPFRMAAATANC-AKIVEY
Guaymas enrichment clone LCA_323	HG764710	FECIRQGLGYPSMRN-DPILIQNAMHWH-------G-HP-LEEA-RSWVHQACMSPCPPTKHGAQPMRMASATANS-AKMVEY
Guaymas enrichment clone LCA_537	HG764709	FECIRQGLGYPSMRN-DPILVHNAMHWH-------G-HP-LEEA-RLWVHQACMSPCPTTKHGTQPMRMASATAKC-AKIVEY
Gowanus Canal petroleum contaminated aquifer clone_OTU3	ADJ51089	FECIRXGLGYPSMRN-DPILISNSMSWH-------G-HP-IEEA-RTWVHQACMSPAPTTKHGFQPMRMANATVNC-AKIVEY
Oil sands tailing SCADC under methanogenic conditions clone_SCADC_assA4	KF995717	FECIRHGLGYPSMRN-DPILIENTVYWY-------G-HP-IEEA-RTWVHQACMSPCPTTKHGFQPFRMASATANT-AKMIEY
Oil sands tailing SCADC under methanogenic conditions clone_SCADC_assA5	KF995716	FECLRHGLGYPNMRN-DPVLIENAMYWH-------G-HP-LEEA-RTWVTQSCMSPCPTTKYGGQPFRMASATANM-AKIVEY
Oil sands tailing SCADC under methanogenic conditions clone_SCADC_assA3	KF995715	FECIRQGLGYPSMRN-DPVLIANSMNWH-------G-HP-IEEA-RTWVHQACMSPCPTTKRGFQPMRMASATANC-AKIMEY
Peptococcaceae sp. SCADC	KFI38250	FECIRHGLGYPNIRH-DDVLVQSNMYWS-------G-TP-LEEA-RTWTAQACIVPCPGTKHGVMPARYSASSTL-GSKCVEL
Smithella sp. SCADC	KFO69021	FECIRQGLGYPSMRN-DPLLIANSMHWH-------G-HP-IEEA-RTWVHQACMSPCPTTKRGFQPMRMASATANC-AKIIEY
Desulfosarcina sp. BuS5 [scaffold_2.3]	2513990058 A39WDRAFT_00284	FDCIRHGLGYPNIRN-DQVLIKANMFWS-------N-TP-EEEA-RTWVAQACIVPAPETKHGCMPMRYSSCTTL-GSKCMEL
Desulfatibacillum aliphaticivorans DSM 15576 [scaffold00017.17]	G491DRAFT_03718	FECIRHGLGYPSIRN-DPLLIQNAMHWH-------G-HP-LEEA-RTWVHQACMSPCPTTKHGVQPMRMASATANC-AKMVEY
Candidatus Magnetoglobus multicellularis Araruama	ATBP01000388	FECIRQGLGYPSMRN-DPILIANAMHWH-------G-HP-IEEA-RLWLHQACMSPCPPTKNGFQPMRMASATANC-AKIIEY
Santa Barbara crude oil polluted sediments metagenome 7	JGI24724J26744_100068502	FECIRHGLGYPSMRN-DSVLIPNAMHWH-------G-HP-LEEA-RQWVHQACMSPCPPTKYGSQPFRMAGTSPNT-SKWIEY
Santa Barbara crude oil polluted sediments metagenome 7	JGI24724J26744_100088922	FDCIRHGLGYPSIRN-DSVLIPNAMYWH-------G-HP-LEEA-RQWVHQACMSPCPPTKYGSQPFRMASAAGNC-AKMAEY
Santa Barbara crude oil polluted sediments metagenome 7	JGI24724J26744_100159401	FECIRHGLGYPNIRN-DSILIPNAMYWH-------G-HP-LEEA-RQWVNQACMSPCPPTKYGCQPNRMAVGL-NC-AKMIEY
Santa Barbara crude oil polluted sediments metagenome 7	JGI24724J26744_100159402	FECIRHGLGYPNIRN-DSVLIPNAMYWH-------G-HP-LDEA-RQWINQACMSPCPPTKYGCQPMRMAVGA-NC-AKMVEY
Santa Barbara crude oil polluted sediments metagenome 7	JGI24724J26744_100368361	FECIRHGLGYPNIRN-DAVLIPNAMHWH-------G-HP-LEEA-RQWVNQACMSPCPPTKYGCQPNRMASAM-NC-AKMIEY
Santa Barbara crude oil polluted sediments metagenome 7	JGI24724J26744_100523621	FECQRHGLGYPSMRN-DDVLIPNLMHWF-------G-HP-LKEA-RRWLHQACMSPAPDTKYGAPPIRYPGASIASGSKAISL
Santa Barbara crude oil polluted sediments metagenome 7	JGI24724J26744_102735631	FECQRHGLGYPSMRN-DDVLIPNLMHWF-------G-HP-LKEA-RRWVQQACMAPAPDTKYGAPPIRYPGASIFGGSKAVSL
Santa Barbara crude oil polluted sediments metagenome 7	JGI24724J26744_103162581	FECIRQGLGYPSMRN-DPVLIANMMHWH-------K-HP-LKEA-RTWLHQACMSPCPTTKWGCQPCRMASATII-AAKSMEY
Santa Barbara crude oil polluted sediments metagenome 7	JGI24724J26744_107047461	FECIRHGLGYPSIRN-DPVLITNGMYWH-------H-HP-LEEM-RTWVHQACMSPCPTTKFGYQPCRMASATMNS-AKMIEY
Santa Barbara crude oil polluted sediments metagenome 7	JGI24724J26744_100057544	FDCIRQGLGYPSMRN-DPILIANCMNWH-------G-HP-LEEA-RQWVHQACMSPCPSTKHGFQPFRMASATANC-AKIIEY
Santa Barbara crude oil polluted sediments metagenome 6	JGI24723J26617_102231391	FECXRDGLGFPSIKH-DEMNTRQ-LEKF-------G-AT-PGEA-RDWCNVLCMSPGHCGRRKAQKARTEGGGGSFPAKIFEI
Santa Barbara crude oil polluted sediments metagenome 1	JGI24023J19991_100209882	FDCIRQGLGYPSMRN-DPILIANCMNWH-------G-HP-LEEA-RQWVHQACMSPCPSTKHGFQPFRMASATANC-AKIIEY

MasD OTU1	SRA bioproject number 278019	FECIRQGLGYPSMRN-DSVLIPNAMYWH-------G-HP-IEEA-RQWVHLACMSPCPPTKHGALPFRMAASLANT-AKWIEY
MasD OTU2	SRA bioproject number 278019	FECIRHGLGYPSMRN-DSVLIPNAMYWH-------G-HP-LKEA-RQWVHQACMSPCPPTKYGSQPFRMAAGSANC-AKFIEY
MasD OTU3	SRA bioproject number 278019	FECIRHGLGYPSIRN-DSVLIPNQMYWH-------G-HP-LDEA-RQWVHQACMSPRPPTKYGSQPFRMASAAGNC-SKMMEY
MasD OTU4	SRA bioproject number 278019	FECLRHGLGYPSIRN-DSVLIPNTMYWY-------G-HP-LEEA-RGWVVQACMSPIPATKNGSCPFRMAAAASNC-CKMIEY
MasD OTU5	SRA bioproject number 278019	FECIRHGLGYPSIRN-DSVLIPNQMYWH-------G-HP-LDEA-RQWVHQACIVPAPPRNMDLSPFRMASAAGNC-SKMMEY
MasD OTU6	SRA bioproject number 278019	FECIRHGLGYPNIRN-DSVLIPNAMYWH-------G-HP-LDEA-RQWINQACMSPLPPTKYGCQPMRMAVGA-NC-AKMVEY
MasD OTU7	SRA bioproject number 278019	FECIRHGLGYPNIRN-DSVLIPNAMYWH-------G-HP-LEEA-RQWVNQACMSPCPPTKYGCQPNRMAVG-LNC-AKMIEY
MasD OTU8	SRA bioproject number 278019	FECIRHGLGYPNIRN-DSVLVPNMMYWH-------G-HP-LEEA-RQWVNQACMSPCPPTKYGCQPNRMASAA-NT-SKMIEY
MasD OTU9	SRA bioproject number 278019	FECIRQGLGYPSMRN-DPVLIPNAMYWH-------G-HP-LEEA-RQWVHQACMSPCPPDEYGCQPNRMASATANC-AKMMEY
MasD OTU10	SRA bioproject number 278019	FECIRQGLGYPSMRN-DPVLIPNAMYWH-------G-HP-LEEA-RQWVHQACMSPCPPTKYGCQPNRMASAT-NC-AKMMEY
MasD OTU11	SRA bioproject number 278019	FECIRQGLGYPSMRN-DPVLIPNAMYWH-------G-HP-LEEA-RQWVHQACMSPCPPTKYGCQPNRMASAT-NC-AKMMEY
MasD OTU12	SRA bioproject number 278019	FECIRQGLGYPSMRN-DPVLIPNAMYWH-------G-HP-LEEA-RQWCILACH-PAPPTKYGCQPNRMASAT-NC-AKMMEY
MasD OTU13	SRA bioproject number 278019	FECIRHGLGYPSMRN-DPVLISNSMYWY-------G-HP-LEEA-RLWVNQACMSPSPPHKHGFNTFRMASATANC-AKMIEY
MasD OTU14	SRA bioproject number 278019	FECIRHGLGYPSMRN-DPVLISNSMYWY-------G-HP-LEEA-RLWVNQACMSPSPPTSMVFQPFRMASATANC-AKMIEY
MasD OTU15	SRA bioproject number 278019	FECIRHGLGYPSMRN-DPICISNAMYWH-------G-HP-LEEA-RQWQHLACMSPNPPTKHGSLPFRMAAALLNC-AKIIEY
MasD OTU16	SRA bioproject number 278019	FECIRHGLGYPNIKN-DSVLIPNAMYWH-------G-HP-LEEA-RQWVNQACMSPCPPTNMDANPIGWRRRPIAP-RXSNIH
MasD OTU17	SRA bioproject number 278019	FECIRHGLGYPNIKN-DSVLIPNAMYWH-------G-HP-LEEA-RQWVNQACMSPCPPXNMDANPIGWRRRSIAP-RXSNIH
MasD OTU18	SRA bioproject number 278019	FECIRHGLGYPNIRN-DSVLIPNCMYWH-------G-HP-LEES-RQWVNQACMSPCPPTSTVASPTGWRXGSIQP-RXLNIH
MasD OTU19	SRA bioproject number 278019	FECIRHGLGYPSMRN-DPILVANAMNWH-------G-HP-IEEA-RTWVHQACMSPVQLQSMASSLYEWPRQPRIA-LKSIEY
MasD OTU20	SRA bioproject number 278019	FECIRQGLGYPSMRN-DPVLVQNAMHWH-------G-HP-LEET-RLWVHQACMSPCPPTKHGNQPFAWRLPLPTA-RRSSNT
MasD OTU21	SRA bioproject number 278019	FECIRQGLGYPSMRN-DPVLVQNAMHWH-------G-HP-LEEA-RLWVHQACMSPCPPRNTGFSLSVWLLQRPTA-RRSSNT
MasD OTU22	SRA bioproject number 278019	FECIRHGLGYPSMRN-DPILVANAMNWH-------G-HP-IEEA-RTWVHQACMSPCPTTKHGFQPIRMAWQPRIA-LRXXST
MasD OTU23	SRA bioproject number 278019	FECIRQGLGYPNIRH-DPIMVANAMHWH-------G-HP-IEEA-RLWVNQACMSPCPPTKHGQQPMRMASATANC-AKIIEY
MasD OTU24	SRA bioproject number 278019	FECIRHGLGYPTLRN-DPILIANAMYWH-------G-HP-IEEA-RTWVHQACMSPCPTTKHGAQPQRMAAATANSTAKAHRG
MasD OTU25	SRA bioproject number 278019	FECIRHGLGYPAMRN-DPILIANAMHWH-------G-HP-IEEA-RTWVHQACMSPCPHTKHGTQPMRMASATANC-AKIIEY
MasD OTU26	SRA bioproject number 278019	FECIRHGLGYPAMRN-DPILISNAMHWH-------R-HP-IEEA-RTWVHQACMSPCPTTKHGTQPMRMAHATANC-AKIMEY
MasD OTU27	SRA bioproject number 278019	FECIRHGLGYPTLRN-DPILVANAMYWH-------G-HP-IEEA-RTWVHQACMSPCPTTKHGTQPMRMAAATANSTAKAHRG
MasD OTU28	SRA bioproject number 278019	FECIRHGLGYPSMRN-DPILVANAVNWH-------G-HP-VKEA-RTWVHQACMSPCPTTKHGFQPFRMASATANC-AKMIEY
MasD OTU29	SRA bioproject number 278019	FECIRHGLGYPSMRN-DPILVHNAMHWH-------G-HP-LEEA-RLWVHQACMSPCPTTKHGFQPFRMASATANC-AKMIEY
MasD OTU30	SRA bioproject number 278019	FECIRQGLGYPSMRN-DPILIANAMSWH-------G-HP-IEEA-RTWVHQACMSPAPTTKHGFQPMRMANATVNC-AKXRRI
MasD OTU31	SRA bioproject number 278019	FECIRHGAGIPFHAN-DPHPYANAVNWH-------A-HP-IEEA-RTWVHQACMSPAPTTKHGFQPMRMASATANC-AKMLQD
MasD OTU32	SRA bioproject number 278019	FECIRHGLGYPSMRN-DPILVANAVNWH-------G-HP-VKEA-RTWVHQACMSPCPTTNTGFQPFRMASATANC-AKMVEY
MasD OTU33	SRA bioproject number 278019	FECIRHGLGYPTLRN-DPILIANAMYWH-------G-HP-IEEA-RTWVHQACMSPCPPTKHGAQPQRMAAATANSTAKAHRG
MasD OTU34	SRA bioproject number 278019	FECIRHGLGYPTLRN-DPILVANAMYWH-------G-HP-IEEA-RTWVHQACMSPCPTTKQGTQPMRMATATANSTAKAHRG
MasD OTU35	SRA bioproject number 278019	FECIRHGLSYPTLRN-DPVLVANAMYWH-------G-HP-IEEA-PHPGFTPCMSPCPTTKHGTQPMRMAAATANS-AKTDRI
MasD OTU36	SRA bioproject number 278019	FECIRQGLGYPAIRN-DPILVHNAMHWH-------G-HP-LEEA-RlWIHQACMSPCPPTKHGNQPFRMASATANC-AKIVEY
MasD OTU37	SRA bioproject number 278019	FECIRQGLGYPSMRN-DPVLVQNAMHWH-------G-HP-LEEA-RLWVHQACMSPCPPTEHGFQPFRMASATANC-AKIVEY
MasD OTU38	SRA bioproject number 278019	FECIRHGLGYPSIRN-DPVLVQNAMHWH-------G-HP-LEEA-RLWVHQACMFPCPTTKHGAQPFRMASATANC-AKIVEY
MasD OTU39	SRA bioproject number 278019	FECIRHGLGYPSMRN-DPILVQNAMHWH-------G-HP-LEEA-RTWVHQACMSPAPTTKHGFQPMRMASATANC-AKIVEY
MasD OTU40	SRA bioproject number 278019	FECIRHGLGYPSMRN-DPILVQNAMHWH-------G-HP-LEEA-RTWVHQACMSPAPTTKHGFQPDRMASATANC-AKIVEY
MasD OTU41	SRA bioproject number 278019	FECIRQGLGYPSMRN-DPILVHNAMHWH-------G-HP-LEEA-RAWVHQACMSPCPDNKHGTQPMRMASATANC-AKIVEY
MasD OTU42	SRA bioproject number 278019	FECIRQGLGYPSMRN-DPILVHNAMHWH-------G-HP-LEEA-RLWVHQACMSPCPTTNTGXQPMRMASATANC-AKIVEY
MasD OTU43	SRA bioproject number 278019	FECIRQGLGYPSMRN-DPILIENAMHWH-------G-HP-LEEA-RTWVHQSCMSPCPTTKNGAQPMRMAAATANC-AKIIEY
MasD OTU44	SRA bioproject number 278019	FECIRQGLGYPSMRN-DPVLIANAMHWH-------K-HP-LEEA-RTWVHQACMSPCPTTKHGAQPFRMASATANT-AKDRHT
MasD OTU45	SRA bioproject number 278019	FECLRQGLGYPSMRN-DPILIENAMHWH-------G-HP-LEEA-RTWVHQSCMSPCPTTKNGVQPMRMAAATANC-AKIVEY
MasD OTU46	SRA bioproject number 278019	FECIRHGLGYPNIRN-DQLLIDNGMYWH-------G-HP-LKEM-RNWVNQACMSPCPQTKYGCQPFRMASCTANT-SKIVEY
MasD OTU47	SRA bioproject number 278019	FECIRHGLGYPSMRN-DPVLIANAMHWH-------K-HP-LEEA-RTWVHQACMSPCPTTKHGAQPFRMASATANT-AKIVEY
MasD OTU48	SRA bioproject number 278019	FECIRHGLGYPSIRH-DPVLIANGMYWH-------N-HP-LKEM-RTWVHQACMSPAPATKHGFQPCRMASATLNC-SKXIEY
MasD OTU49	SRA bioproject number 278019	FECIRHGLGYPSIRH-DPVLIANGMYWH-------N-HP-LKEM-RTWVHQACMSPAPTTKHGFQPCRMASATLNC-SKMVEY
MasD OTU50	SRA bioproject number 278019	FECIRQGLGYPSIRN-DPVLIANGMYWH-------G-HP-LEEM-RLWVHQACMSPNPPTKLGFQPCRMASXTMNT-SKMIEY
MasD OTU51	SRA bioproject number 278019	FECIRQGLGYPSMRN-DPILINNGMYWH-------H-HP-LEEM-RTWVHQACMSPCPATKXGHQPCRMASATMNS--KMIEF
MasD OTU52	SRA bioproject number 278019	FECIRQGLGYPSIRN-DPILINNGMHWH-------H-HP-LEEM-RNWVHQACMSPNPTTKHGLQPCRMASATANT-EKAIEY
MasD OTU53	SRA bioproject number 278019	FECIRQGLGYPSMRN-DPVLIANGMYWH-------G-HP-LEEM-RTWVHQACMSPCPTTKYGYQPCRMASATMNT-AKMIEY
MasD OTU54	SRA bioproject number 278019	FECIRQGLGYPSMRN-DPVLIANGMYWH-------G-HP-LEEM-RTWVHQACMSPCPHTKYGYQPCRMASATMNT-AKMIEY
MasD OTU55	SRA bioproject number 278019	FECIRQGLGYPSMRN-DPVLVQNAMHWH-------G-HP-LEEA-RLWVHQACMSPCPPTKHGNQPFRMASATANC-AKIVEY
MasD OTU56	SRA bioproject number 278019	FECIRHGLGYPSMRN-DPVCVENTVHWY-------G-HP-LEEA-RTWVHQACMSPCPTTKHGFQPMRMASATANC-AKMVEY
MasD OTU57	SRA bioproject number 278019	FECIRHGLGYPAMRN-DPILIANAMHWH-------G-HP-IEEA-RTWVHQACMSPQPPTKYGFQPCRMASATMNT-SKMIEY
MasD OTU58	SRA bioproject number 278019	FECIRHGLGYPSMRN-DPICVENTVHWY-------G-HP-LEEA-RTWVHQACMSPCPTPQHGFQPMRMASATANT-AKMIEY

MasD OTU59	SRA bioproject number 278019	FECIRHGLGYPSMRN-DPIPVENTVYWY-------G-HP-LEEA-RTWVHQACMSPCPTTKHGFQPMRMASATANC-AKMVEY
MasD OTU60	SRA bioproject number 278019	FECIRHGLGYPSIRN-DDVLIPNIMHWF-------G-HP-PEGS-EALVTPGCHGTSSGYKMGC-SISAGTP-RPL-XXQAQK
MasD OTU61	SRA bioproject number 278019	FECIRHGLGYPSMRN-DDVLIPNLMHWF-------G-HP-LKEA-RRWGTPGCYGACTGYEVGSAGLEVSAA-LNN-RRVKGC
MasD OTU62	SRA bioproject number 278019	FECIRHGLGYPSIRN-DDVLIPNLMHWF-------G-HP-LKEA-RRWVHQACMAPAPDTKWAAPALRYPQ-------PSIAT
MasD OTU63	SRA bioproject number 278019	FECQRHGLGYPSMRN-DDVLIPNLMHWF-------G-HP-LKEA-RRWVQQACMAPAPDTKYGAPPIRYPGASISS-GKAISL
MasD OTU64	SRA bioproject number 278019	FECIRHGLGYPSMRN-DDVLIPNLMHWF-------G-HP-LKEA-RRWVHQACMAPAPDTKWAAPALRYPQP-SII-GGSKAV
MasD OTU65	SRA bioproject number 278019	FECIRHGLGYPSMRN-DDVLIPNLMALV-------W-SS-IEGG-KALGTPGCYGACTXYEVGSAGLEVSAA-LDN-RRSKAV
MasD OTU66	SRA bioproject number 278019	FECIRHGLGYPSIRN-DDVLIPNIMHWF-------G-HP-LKEA-RRWLHQACMAPAPDTKWGAPSLRYPHG-LYN-NRLKSD
MasD OTU67	SRA bioproject number 278019	FECIRHGLGYPSMRN-DDVLIPNLMHWF-------G-HP-LKEA-RRWVHQACMAPAPDTKWAAPALRYPQP-SII-GGSKAV
MasD OTU68	SRA bioproject number 278019	FECIRHGLGYPAMRN-DPILISNAMHWH-------R-HP-IEEA-RTWVHQACMSPCPTTKHGTQPMRMAHATANC-AKIMEY
MasD OTU69	SRA bioproject number 278019	FECIRHGLGYPSMRN-DDVLIPNLMHWF-------G-HP-LKEA-RRWVHQACMAPAPDTKWAAPALRYPQPSIATGSKAVSM
MasD OTU70	SRA bioproject number 278019	FECIRHGLGYPSMRN-DPVLISNSMYWY-------G-HP-LEEA-RLWVNQACMSPSPPTKHGAQPFRMASATANC-AKIVEY
MasD OTU71	SRA bioproject number 278019	FECIRHGLGYPSIRN-DDVLIPNIMHWF-------G-HP-LKEA-RRWVHQACMAPAPDTKWGAPSLRYPQA-SIA-TGSKAI
MasD OTU72	SRA bioproject number 278019	FECLRQGLGYPSFQH-NDICISILTSHW-------G-AT-IEEA-RSWANVVCMSPGVMKGRGGQGVRYASWMMVG-TKPLEL
MasD OTU73	SRA bioproject number 278019	FECIRQGLGYPSMRN-DPILVHNAMHWH-------G-HP-LEEA-RLWVHQACMSPCPTTKHGAQPMRMASATANC-AKIVEY
MasD OTU74	SRA bioproject number 278019	FECIRHGLGYPSIRN-DPVLVQNAMHWH-------G-HP-LEEA-RLWVHQACMSPCPTTKHGAQPFRMASATANC-AKIVEY
MasD OTU75	SRA bioproject number 278019	FECIRHGLGYPSIRN-DPVLVQNAMHWH-------G-HP-LEEA-RLWVHQACMSPCPTTRHGVQPFRMAAATANC-AKIVEY
MasD OTU76	SRA bioproject number 278019	FECIRQGLGYPSMRN-DPILVHNAMHWH-------G-HP-LEEA-RLWVHQACMSPCPTTKHGTQPMRMASATANC-AKIVEY
MasD OTU77	SRA bioproject number 278019	FECIRQGLGYPSMRN-DPILVHNAMHWH-------G-HP-LEEA-RLWVHQACMSPCPTTKHGAQPDRMASATANC-AKIVEY
MasD OTU78	SRA bioproject number 278019	FECIRQGLGYPSMRN-DPVLVQNAMHWH-------G-HP-LEEA-RLWVHQACMSPCPPTKHGYQPFRMASATANC-AKIVEY
MasD OTU79	SRA bioproject number 278019	FECIRQGLGYPSMRN-DPILVHNAMHWH-------G-HP-LEEA-RLWVHQACMSPCPRQX-GAQPMRMAAATANC-AKIVEY
MasD OTU80	SRA bioproject number 278019	FECIRQGLGYPNIRH-DPIMVANAMHWH-------G-HP-IEEA-RLWVNQACMSPCPPTKHGQQPMRMASATANC-AKIIEY
MasD OTU81	SRA bioproject number 278019	FECIRQGLGYPNIRH-DPIMVANAMHWH-------G-HP-IEEX-RLWVNQACMSPCPPTKHGQQPMRMASATANC-AKIIEY
MasD OTU82	SRA bioproject number 278019	FECIRQGLGYPSMRN-DPILIANAMSWH-------G-HP-IEEA-RTWVHQACMSPAPTTKHGFQPMRMANATVNC-AKIVEY
MasD OTU83	SRA bioproject number 278019	FECIRQGLGYPSMRN-DPVLVQNAMHWH-------G-HP-PEEA-RLWVHQACMSPCPPTKHGNQPFRMASATANC-AKIVEY

Table S2 GeneFISH polynucleotide probes developed for the detection of *masD*. Probe length: 351 bp (*masD* position 1487 – 1838, *Aromatoleum* sp. OcN1).

Target	Probe type	No. of probes	*masD*-carrying clone for probe synthesis	Accession numbers	ΔT_m [°C]	Conc. [ng µl^{-1}]
Cluster Ia-g	dsDNA	1	GB SCA-incubations clone_24	HG764728	-	171
	dsDNA	3	GB SCA-incubations clone_32	HG764722	5.09*	162
			GB SCA-incubations clone_48	LN610418		163
			GB SCA-incubations clone_55	LN610421		171
Cluster II	dsDNA	1	GB SCA-incubations clone_40	LN610415	-	76
Cluster III	dsDNA	4	GB SCA-incubations clone_43	LN610417	2.71*	120
			GB SCA-incubations clone_50	HG764720		129
			GB SCA-incubations clone_51	LN610419		116
			GB SCA-incubations clone_61	LN610423		41

* T_m calculated according to Wetmur, J.G., and Fresco, J. (1991) DNA Probes: Applications of the principles of nucleic acid hybridization. Critical Reviews in Biochemistry and Molecular Biology 26: 227-259.

Stagars et al.

Table S3. MasD OTU abundance on different taxonomic levels found at investigated hydrocarbon seep sites.

Sample ID	Quality reads	$OTU_{0.96}$ no.[a]	$OTU_{0.86}$ no.[b]	$OTU_{0.72}$ no.[c]
GoM140	430	58	33	16
GoM13	487	47	24	12
GoM17	226	27	19	11
GoM4463	154	44	24	15
GoM156	803	87	45	20
GB4573	989	93	46	24
GB4484	572	66	37	17
AMV760	134	21	13	10
AMV825	376	50	28	15
HR19	65	13	8	4
NZ315	722	65	33	21
Tomm	173	33	21	11
Total	5131	420	178	83

OTU definition on [a] species level; [b]genus level; [c] family level

Table S4. Relative single sequence OTU (SSO_{rel}) found in the MasD dataset retrieved from the 12 investigated hydrocarbon seep sites. SSO_{rel} are defined as $OTU_{0.96}$ that occurred only once in one of the sample, but might occur more often in one or more of the other samples. Numbers given are quality read numbers retrieved from the individual sites.

	OTU2	OTU3	OTU4	OTU5	OTU6	OTU7	OTU8	OTU9	OTU10	OTU11	OTU12	OTU14	OTU15	OTU16	OTU17	OTU18	OTU19	OTU20	OTU21	OTU22	OTU25	OTU27	OTU28
**GoM140**	1	5	0	0	1	0	161	0	0	0	0	0	56	2	0	1	0	0	0	0	29	0	0
**GoM13**	5	216	0	0	0	21	0	0	71	1	0	14	1	0	0	5	0	5	0	27	0	0	1
**GoM17**	1	0	11	2	2	0	1	2	5	50	0	0	1	0	0	0	0	0	0	0	1	0	0
**GoM4463**	6	4	1	0	1	21	2	2	0	0	0	7	3	0	0	18	0	2	0	0	1	8	0
**GoM156**	7	205	0	0	1	142	8	0	0	0	129	0	8	66	0	9	0	30	0	2	2	4	0
**GB4573**	6	0	5	246	194	0	0	0	0	0	0	0	0	0	60	0	0	1	0	0	0	1	0
**GB4484**	10	0	273	1	0	0	0	1	0	2	0	13	0	1	1	0	0	0	47	1	0	5	0
**AMV760**	1	1	0	0	0	0	1	0	0	87	1	21	1	0	0	0	0	0	0	0	0	0	0
**AMV825**	10	54	0	0	1	12	1	0	32	2	0	12	1	1	0	3	0	12	1	10	0	3	0
**HR19**	34	0	0	0	0	0	0	8	1	0	0	0	0	0	0	2	1	0	0	0	0	3	0
**NZ315**	385	0	19	10	0	0	1	85	33	1	0	1	1	0	0	15	52	0	0	0	0	3	24
**Tomm**	38	0	0	1	1	1	2	58	4	0	0	8	1	1	0	7	0	0	0	1	0	1	0
**SUM**	504	485	309	260	201	197	177	156	146	143	130	76	73	71	61	60	53	50	48	41	33	28	25

	OTU29	OTU31	OTU36	OTU37	OTU40	OTU42	OTU44	OTU46	OTU48	OTU49	OTU50	OTU52	OTU53	OTU56	OTU61	OTU66	OTU67	OTU69	OTU78	OTU79	OTU80	OTU81	OTU82
**GoM140**	0	4	0	3	10	10	1	0	0	0	0	1	0	4	4	0	3	0	0	0	0	0	0
**GoM13**	0	0	1	0	0	0	3	0	0	0	0	4	1	0	0	0	0	0	0	0	0	0	0
**GoM17**	0	0	0	0	0	0	0	0	0	0	0	0	0	0	0	0	0	0	0	1	0	3	3
**GoM4463**	0	3	0	4	1	0	0	0	0	0	0	0	2	1	1	1	0	3	0	0	0	0	0
**GoM156**	18	11	9	4	0	1	5	0	6	0	0	1	3	0	0	3	0	0	0	0	1	0	0
**GB4573**	0	1	0	0	0	0	0	1	0	0	1	0	0	0	0	0	1	0	0	0	0	0	0
**GB4484**	1	0	0	0	0	0	0	7	0	0	0	0	0	0	0	0	0	0	0	1	0	0	0
**AMV760**	0	0	0	0	0	0	0	0	0	6	0	0	0	0	0	0	0	0	0	0	0	0	1
**AMV825**	0	0	3	0	0	0	0	0	1	1	0	0	0	0	0	0	0	0	0	0	0	1	0
**HR19**	0	0	0	0	0	0	0	0	0	0	0	0	0	0	0	0	0	0	0	0	0	0	0
**NZ315**	1	0	0	0	0	0	0	0	0	0	0	0	0	0	0	0	0	0	3	1	0	0	0
**Tomm**	0	0	0	1	0	0	0	0	0	0	5	0	0	0	0	0	0	1	1	1	3	0	0
**SUM**	20	19	13	12	11	11	9	8	7	7	6	6	6	5	5	4	4	4	4	4	4	4	4

	OTU83	OTU86	OTU88	OTU89	OTU91	OTU92	OTU97	OTU101	OTU102	OTU103	OTU110	OTU111	OTU120	OTU126	OTU128	OTU141	OTU145	OTU146	OTU149	OTU152	OTU153	OTU155
**GoM140**	0	2	0	0	0	0	0	0	0	0	0	0	0	0	0	0	0	1	0	1	1	0
**GoM13**	3	0	0	0	0	0	0	0	0	1	1	0	0	0	1	0	1	0	1	0	0	1
**GoM17**	0	1	0	0	0	2	0	0	0	0	1	1	0	0	0	0	0	0	0	0	0	0
**GoM4463**	0	0	0	0	0	0	0	1	0	0	0	0	0	0	0	0	0	1	0	1	0	0
**GoM156**	1	0	0	0	0	0	0	0	0	0	0	0	0	1	0	1	0	0	0	0	1	0
**GB4573**	0	0	0	2	0	0	2	0	0	0	0	0	0	0	1	0	0	0	0	0	0	0
**GB4484**	0	0	2	1	1	0	1	2	0	0	0	0	0	0	0	0	0	0	0	0	0	0
**AMV760**	0	0	0	0	0	0	0	0	0	0	0	0	0	0	0	1	0	0	0	0	0	0
**AMV825**	0	0	1	0	2	1	0	0	0	2	0	1	1	0	0	0	1	0	1	0	0	1
**HR19**	0	0	0	0	0	0	0	0	1	0	0	0	0	1	0	0	0	0	0	0	0	0
**NZ315**	0	0	0	0	0	0	0	0	2	0	0	0	1	0	0	0	0	0	0	0	0	0
**Tomm**	0	0	0	0	0	0	0	0	0	0	0	0	0	0	0	0	0	0	0	0	0	0
**SUM**	4	3	3	3	3	3	3	3	3	3	2	2	2	2	2	2	2	2	2	2	2	2
